# Detecting hierarchical organization of pervasive communities by modular decomposition of Markov chain

**DOI:** 10.1038/s41598-022-24567-x

**Published:** 2022-11-23

**Authors:** Hiroshi Okamoto, Xule Qiu

**Affiliations:** 1 Department of Bioengineering, The University of Tokyo, Tokyo, 113-8656 Japan; 2 DWANGO Co., Ltd., Tokyo , Japan; 3 FUJIFILM Business Innovation Corp., Tokyo, Japan

**Keywords:** Complex networks, Computational neuroscience

## Abstract

Connecting nodes that contingently co-appear, which is a common process of networking in social and biological systems, normally leads to modular structure characterized by the absence of definite boundaries. This study seeks to find and evaluate methods to detect such modules, which will be called ‘pervasive’ communities. We propose a mathematical formulation to decompose a random walk spreading over the entire network into localized random walks as a proxy for pervasive communities. We applied this formulation to biological and social as well as synthetic networks to demonstrate that it can properly detect communities as pervasively structured objects. We further addressed a question that is fundamental but has been little discussed so far: What is the hierarchical organization of pervasive communities and how can it be extracted? Here we show that hierarchical organization of pervasive communities is unveiled from finer to coarser layers through discrete phase transitions that intermittently occur as the value for a resolution-controlling parameter is quasi-statically increased. To our knowledge, this is the first elucidation of how the pervasiveness and hierarchy, both hallmarks of community structure of real-world networks, are unified.

## Introduction

Discovering modular structure of networks, a process known as community detection, is fundamental for understanding functional properties of complex systems described by networks. Much effort has been dedicated to developing effective and efficient methods for community detection since the early days of network science, rendering hundreds of methods proposed so far^1^. Meanwhile, pursuing the universal method for community detection is criticized as ill-defined^2^, as individual methods are separately optimized to find what they respectively define as communities. To make our study of community detection meaningful, therefore, we should specify what we seek as communities. On this account, we begin by classifying potential structure of communities into two categories; one would be described as ‘definite’ and the other as ‘pervasive’.

‘Definite’ communities are characterized as having clear boundaries that separate their inside from their outside. Whether a given node belongs to a specific community is clearly determined by its boundary. Each node (or link) may belong to more than one community. If this is the case, communities are overlapping^3–8^. Even so, clear boundaries still exist, though they are intersecting. In contrast, ‘pervasive’ communities are best characterized by the absence of clear boundaries. More precisely, a pervasive community can be defined by a conditional probability *p*(*n*|*k*), which represents the relative membership of node *n* in community *k*, and a probability $\pi (k)$, which corresponds to the relative size of community *k*. The *p*(*n*|*k*) generally takes graded values. Pervasive communities therefore have distributed structure, making separation of their inside and outside obscure. Rather, any node can be a member of any pervasive community irrespective of the level of its membership in that community. In the begging of the next section, we will discuss the notion that a common process of networking in social and biological systems naturally leads to pervasive structure of communities.

**Figure 1:**
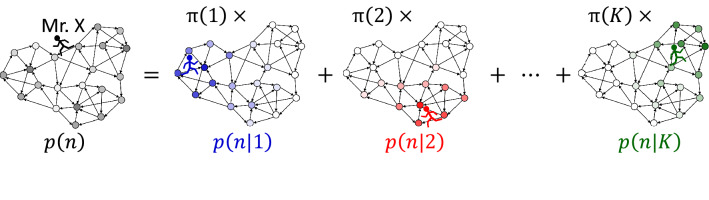
Illustrating the image of MDMC: $p(n) = \sum _{k=1}^{K} \pi (k) p(n|k)$. The probability *p*(*n*) that Mr. X is at node *n* is expressed by the grey strength of each node in the network drawn in the left-hand side. The probability $p(n|k)\ (k=1, 2,\ \ldots ,\ K)$ that Mr. X is at node *n* conditioned that he is staying in community *k* is expressed by the colour strength of each node in the networks drawn in the right-hand side. The $\pi (k)$ is the probability that Mr. X is staying in community *k*.

Most of community detection methods proposed so far are designed to discover definite structure of communities^1^. Although several related attempts have already been examined^9–13^, to establish theoretically well-principled detection of pervasive communities is still a challenge of network science (see also “Discussions” for our view about soft clustering using graph embedding). Here we put forward a mathematical formulation to detect communities as pervasively structured objects. Our key idea is to express a random walk spreading over the entire network as a mixture of localized random walks (Fig. 1). By the use of a probabilistic machine-learning approach to mixture modelling^14^, we have succeeded in formulating decomposition of the global random walk into local modules as a proxy for pervasive communities.

Hierarchical organization of communities is another essential aspect of real-world networks^5,7,15–23^. As noted by Simon^24^, the hierarchy in modular structure is a fundamental architecture of social and biological systems. To fully illustrate the hierarchy, it is necessary not only to discover modules (e.g., clusters or communities) in each layer but also to identify parent–child relationships between modules in adjacent layers. Parent–child relationships in hierarchical organization of definite communities can be identified just by finding communities to which individual nodes belong in each layer and then tracing the affiliation of each node from one layer to the next.

Turning now to pervasive communities, we realize that little has been discussed about their hierarchical organization so far. Given that the hierarchy and pervasiveness are both hallmarks of community structure of real-world networks, it may be surprising that few scholars have attempted to unify them, albeit several studies conducted to merge the hierarchy and overlapping of definite communities^5,7,23,25^. A difficulty lies in that, since pervasive structure no longer defines a single or a finite number of communities to which a given node belongs, the tracing strategy to identify parent–child relationship as above is no longer effective. To reveal hierarchical organization of pervasive communities is therefore another huge challenge of network science. Towards this ultimate goal, here we demonstrate that hierarchical organization of pervasive communities can be unveiled by exploiting phase transition. To our knowledge, this is the first elucidation of hierarchical organization of pervasive communities.

## Results

### Relative membership of nodes

We assume that non-negative quantities $f_k (n)\ (k=1,\ \ldots ,\ K)$ underlie the network from which we wish to detect communities. In the social context, $f_k (n)$ represents, for instance, the frequency of participation or the level of commitment of person *n* in activity *k*; in the brain, it would be the firing rate of neuron *n* during the performance of cognitive behaviour *k*. We further assume a non-negative quantity *g*(*k*), which would represent the frequency of occurrence of activity *k*. Just for convenience, we normalize $f_k (n)$ and *g*(*k*) so that we can manipulate them as probabilities: $p(n|k) \equiv {f_k(n)}/{\sum _n f_k(n)} \ (\ge 0)$ and $\pi (k) \equiv {g(k)}/{\sum _k g(k)} \ (\ge 0)$, hence $\sum _n p(n|k) =1$ and $\sum _k \pi (k) =1$. We call *p*(*n*|*k*) the ‘relative membership’ of node *n* in group *k* and $\pi (k)$ the ‘relative size’ of group *k*. The relative membership should not be confused with the membership function $m_k(n)$ of fuzzy set *k*^26,27^, though they looks similar; in fact, they are strictly different concepts. The $m_k(n)$ is the absolute level of belonging of element *n* to set *k*, ranging from 0 to 1, and is defined independently from those for the other nodes and the other sets. Indeed, we cannot derive $m_k(n)$ from *p*(*n*|*k*) and $\pi (k)$, and vice versa.

### Connecting co-appearing nodes

The probability of co-appearance of nodes *n* and *m* in group *k* is therefore given by *p*(*n*|*k*)*p*(*m*|*k*). As the probability of occurrence of group *k* is $\pi (k)$, the probability of co-appearance of nodes *n* and *m* in any group is given by $\sum _k \pi (k)p(n|k)p(m|k)$. We assume that connections between nodes *n* and *m* are generated according to this probability; specifically, the number (*a*) of links will be generated between nodes *n* and *m* following the Poisson distribution $p(a) = \mu ^a e^{-\mu } / a!$ with the rate parameter $\mu \propto \sum _k \pi (k) p(n|k) p(m|k)$. Connecting co-appearing nodes is a common process of generating social and biological networks. For instance, co-participation of persons in any activity is the chance for them to become acquainted and get connected on social networking service. The process is also comparable to Hebb’s rule^28^, which is the leading principle of modern neuroscience. This rule states that co-activation of neurons strengthens the synaptic connection between them; more succinctly, cells that fire together wire together.

### Definition of community detection

Nodes that co-appear in group *k* become densely connected, thus forming a structure called community *k*. Therefore, *p*(*n*|*k*) and $\pi (k)$ represent the relative level of membership of node *n* in community *k* and the relative size of community *k*, respectively. Now we specify our problem setting of community detection. Here we define community detection as a task to decode *p*(*n*|*k*) and $\pi (k)$ from a given network (namely, the adjacency matrix determining this network). In doing so, we assume that the network from which we wish to detect communities is generated through probabilistic linking between co-appearing nodes as described above.

### Definite versus pervasive community

Suppose $\pi (k)>0$; that is, community *k* is non-vanishing. Then, possible patterns of *p*(*n*|*k*) as a function of *n* can roughly be divided into two categories. In the first category, $p(n|k)>0$ for some *n*’s ($n \in {\mathcal {N}}_k$) and $p(n|k)=0$ for the other *n*’s ($n \notin {\mathcal {N}}_k$); typically, $f_k(n)=1$ and hence $p(n|k) = 1 / \left| {\mathcal {N}}_k \right|$ for $n \in {\mathcal {N}}_k$ (node *n* is a member of community *k*), and $f_k(n)=0$ and hence $p(n|k) = 0$ for $n \notin {\mathcal {N}}_k$ (node *n* is a non-member of community *k*). Patterns of *p*(*n*|*k*) in this category give clear separations of nodes between members and non-members. Therefore, community *k* has a clear boundary that definitely separate its inside and outside. We will refer to communities with such clear boundaries as ‘definite’ communities. Specially, the problem of detecting definite communities can be replaced to that of finding their boundaries, which can be solved with less computational cost than directly solving *p*(*n*|*k*) and $\pi (k)$. Most of community detection methods proposed so far are designed to find such boundaries^1^.

In the second category, *p*(*n*|*k*) is nonzero positive for any node *n*. Indeed, this is often the case in the social context, where persons might be unaware to which activities they are participating or even ignorant about segmentation of activities itself. In such situations, each person would contingently participate in any activity even if the level of his/her participation to it might be relatively low. Consequently, *p*(*n*|*k*) would in principle have any positive value. Therefore, community *k*, in which the relative level of membership of each node is represented by $p(n|k)>0$, has no clear boundary separating member and non-member nodes; rather, every node is a member of any community irrespective of the level of membership in this community. We will refer to such communities as ‘pervasive’ communities. The present study seeks to find and evaluate methods that are optimized to find pervasive communities. Unlike detection of definite communities, that of pervasive communities has no other way than to directly solve *p*(*n*|*k*) and $\pi (k)$.

Contingent activation of neuron *n* in response to the execution of cognitive behaviour *k*, which is measured by the firing rate, would be graded rather than all-or-none^29–31^. Therefore, *p*(*n*|*k*) corresponding to the firing rate of neuron *n* is, in principle, nonzero positive. Hebb’s rule thus forms pervasive communities in the brain network, which are called ‘cell assemblies’ in neuroscience^28^. Cell assemblies are hypothesized to be the functional modules of information processing in the brain. Pervasive structure of cell assemblies would be essential for parallel distributed processing in the brain.

Concluding this subsection, we argue that pervasiveness is a hallmark of the structure of communities in real-world networks that are generated not by any global design or control but in a self-organized manner by some local rules such as connecting contingently co-appearing nodes. Artificial boundaries that separate members and non-members would be set only after the formation of a faction is declared (such as the separation of the karate club^32^) or a list of names is established. The absence of each node’s (e.g. each person’s) explicitness to which communities he/she is belonging makes it difficult to define the ground truths of pervasive communities for real-world networks, which would commonly be required to validate community detection.

### Soft overlapping of pervasive communities

Co-existence of a number of pervasive communities in a network entails intricate, soft overlapping between them. Detecting soft-overlapping communities has been perceived as a difficult problem, remaining largely unsolved. In our problem setting of community detection, however, soft overlapping of communities is consequently found once *p*(*n*|*k*) and $\pi (k)$ are solved, though difficulty of the problem is not necessarily reduced with that. In our view, soft-overlapping is not the definition but consequential characteristics of pervasive communities. In this sense, finding soft-overlapping of communities itself is not the goal but a bonus of pervasive community detection.

### Modular decomposition of Markov chain (MDMC)

It is noticeable that prominent methods for community detection are, or can be, based upon the idea of random walk (see Supplementary Information for brief review). The map equation^33–36^, which detects communities by searching for the most parsimonious way to describe a random walk on the network, has performed the best in comparative studies^37,38^. Modularity maximization^39,40^, renowned as a standard for community detection^41^, is equivalent to maximization of the probability that a random walker remains in the same community, relative to that expected under null models^42–44^. These methods suggest that exploiting random walk is an effective approach to designing community detection^45^. Our formulation, which we call modular decomposition of Markov chain (MDMC), is also in line with this approach.

The idea behind MDMC is illustrated in Fig. 1. Imagine that a random walker, say Mr. X, is travelling in a network from which we wish to detect communities. Let *p*(*n*) be the probability that Mr. X is at node *n*. Given that the network comprises several communities, Mr. X is trapped by some community and stays there for a while; at some time, he chances to move to another community and then stays there for a while, and so forth. Let *p*(*n*|*k*) be the probability that Mr. X is at node *n* conditioned that he is staying in community *k*. The ‘global’ probability *p*(*n*) will then be expressed as a mixture of ‘local’ probabilities *p*(*n*|*k*):

1 $$\begin{aligned} p(n) = \sum _{k=1}^{K} \pi (k) p(n|k)\ , \end{aligned}$$

where *K* is the putative number of communities; $\pi (k)\ (\ge 0)$ is the probability that Mr. X is staying in community *k*, satisfying $\sum _{k=1}^{K} \pi (k) =1$; $\pi (k)>0$ if community *k* really exists and $\pi (k)=0$ otherwise. Community detection is achieved if Eq. (1) is solved to *p*(*n*|*k*) and $\pi (k)$.

Imagine that *D* ‘investigators’ are distributed over the network to search for the whereabouts of Mr. X. Each investigator ought to detect which ‘street’ (namely, which link) Mr. X is moving along. Search for Mr. X by individual investigators is carried out independently; that is, they neither communicate nor exchange information with each other. Suppose that investigator *d* has observed that Mr. X is moving along a link from node $n_d^{\textrm{from}}$ to node $n_d^{\textrm{to}}$. Let $\tau ^{(d)}$ denote the result of this observation. The probability of $\tau ^{(d)}$ conditioned that Mr. X is staying in community *k* is modelled by the product of categorical distributions:

2 $$\begin{aligned} p(\tau ^{(d)}|\left\{ p_t(n|k) \right\} _{n=1}^{N}) & = \prod _{n=1}^{N}\left[ p_t(n|k) \right] ^{\delta _{n,\ n_d^{\textrm{from}}}} \times \prod _{n=1}^{N}\left[ p_t(n|k) \right] ^{\delta _{n,\ n_d^{\textrm{to}}}} \nonumber \\ & = p_t(n_d^{\textrm{from}}|k)p_t(n_d^{\textrm{to}}|k)\ , \end{aligned}$$

where *N* is the total number of nodes; and $p_t(n|k)$ is the probability that Mr. X is at node *n* at time *t* conditioned that he is staying in community *k*. The form of Eq. (2) is consistent with the probability that persons *n* and *m* co-appear in activity *k*, which has been discussed earlier in this section to define pervasive communities. Therefore, solving for $p_t(n|k)$ (and $\pi _t(k)$) enables us to detect what we define as communities.

In Eq. (2), $\left\{ p_t(n|k) \right\} _{n=1}^{N}$, which satisfy $p_t(n|k)\ge 0$ and $\sum _{n=1}^{N} p_t(n|k) =1$, serve as parameters. These are then upgraded to stochastic variables by introducing the conjugate prior defined as a Dirichlet distribution in the form

3 $$\begin{aligned} p\left( \left\{ p_t(n|k) \right\} _{n=1}^{N} | \left\{ p_{t-1}(n|k) \right\} _{n=1}^{N} \right) & = \frac{\Gamma \left( \alpha + N \right) }{\prod _{n=1}^{N} \Gamma \left( \alpha \sum _{m=1}^{N} T_{nm} p_{t-1}(m|k) + 1 \right) } \nonumber \\ & \quad \times \prod _{n=1}^{N} \left[ p_t(n|k) \right] ^{ \left( \alpha \sum _{m=1}^{N} T_{nm} p_{t-1}(m|k) +1 \right) - 1}\ , \end{aligned}$$

where $\Gamma (\cdot )$ denotes the gamma function; $T_{nm}$ is the rate for transition from node *m* to node *n*; $\alpha \ (>0)$ is the parameter that controls the concentration of the Dirichlet distribution. As $\alpha \rightarrow +\infty$, the Dirichlet distribution is concentrated onto the point that gives the ‘classical’ Markov chain

4 $$\begin{aligned} p_t(n|k) = \sum _{m=1}^{N}T_{nm} p_{t-1}(m|k)\ . \end{aligned}$$

For finite values of $\alpha$, $p_t(n|k)$ fluctuates around $\sum _{m=1}^{N} T_{nm} p_{t-1}(m|k)$; the smaller the value for $\alpha$, the more apart $p_t(n|k)$ can deviate from $\sum _{m=1}^{N} T_{nm} p_{t-1}(m|k)$. Equation (3) thus describes a stochastic generalization of the Markov chain, which will turn out to be an essential component of MDMC. In the absence of this prior, our formulation becomes equivalent to a class of stochastic block modelling (see “Methods”).

Results of observations by *D* investigators are gathered to give data ${\mathcal {D}}=\left\{ \tau ^{(1)},\ \ldots ,\ \tau ^{(D)} \right\}$. The elements $\tau ^{(1)},\ \ldots ,\ \tau ^{(D)}$ are independent and identically distributed (i.i.d.). Now we introduce latent variables ${\textbf{z}}^{(d)}=\left\{ z_1^{(d)},\ \ldots ,\ z_K^{(d)} \right\}$ representing the community in which Mr. X was staying when investigator *d* observed him. These variables are given by a 1-of-*K* vector (just one component is unity and the $K-1$ others are zero); for instance, if Mr. X is staying in community $k^{\prime }$ when observed by investigator *d*, $z_{k}^{(d)}=\delta _{kk^{\prime }}$. The probability of ${\textbf{z}}^{(d)}$ is modelled by a categorical distribution

5 $$\begin{aligned} p\left( {\textbf{z}}^{(d)}|\left\{ \pi _t(k) \right\} _{k=1}^{K} \right) = \prod _{k=1}^{K}\left[ \pi _t(k) \right] ^{z_{k}^{(d)}}\ , \end{aligned}$$

where $\left\{ \pi _t(k) \right\} _{k=1}^{K}$ are parameters satisfying $\pi _t(k)\ge 0$ and $\sum _{k=1}^{K} \pi _t(k) =1$. Under the i.i.d. assumption for data ${\mathcal {D}}$, the joint probability is expressed as

6 $$\begin{aligned} p\left( {\mathcal {D}},\ {\textbf{P}}_t,\ {\textbf{Z}}|\left\{ \pi _t(k) \right\} _{k=1}^{K} \right) & = \prod _{d=1}^{D}\prod _{k=1}^{K}\left[ \pi (k) \prod _{n=1}^{N} \left[ p_t(n|k) \right] ^{\delta _{n,\ n_d^{\textrm{from}}}+\delta _{n,\ n_d^{\textrm{to}}}} \right] ^{z_{k}^{(d)}}\nonumber \\ & \quad \times \prod _{k=1}^{K} \frac{\Gamma \left( \alpha + N \right) }{\prod _{n=1}^{N} \Gamma \left( \alpha \sum _{m=1}^{N} T_{nm} p_{t-1}(m|k) + 1 \right) } \nonumber \\ & \quad \times \prod _{k=1}^{K}\prod _{n=1}^{N} \left[ p_t(n|k) \right] ^{\alpha \sum _{m=1}^{N} T_{nm}p_{t-1}(m|k)}, \end{aligned}$$

with notations ${\textbf{P}}_t=\left\{ \left\{ p_t(n|1) \right\} _{n=1}^{N},\ \ldots ,\ \left\{ p_t(n|K) \right\} _{n=1}^{N} \right\}$ and ${\textbf{Z}}=\left\{ {\textbf{z}}^{(d)} \right\} _{d=1}^{D}$.

In general, one can solve the joint probability (6) to the stochastic variables ${\textbf{P}}_t$, ${\textbf{Z}}$ and the parameters $\left\{ \pi _t(k) \right\} _{k=1}^{K}$ by optimization procedure. Indeed, one can derive expectation-maximization (EM) algorithm as a solver for this optimization problem (see “Methods” for a detailed derivation), which is given in the form:

*E-step*

7 $$\begin{aligned} r(k|l) = \frac{\pi _{t-1}(k) p_{t-1}(n_{l}^{\textrm{from}}|k) p_{t-1}(n_{l}^{\textrm{to}}|k)}{\sum _{k=1}^{K} \pi _{t-1}(k) p_{t-1}(n_{l}^{\textrm{from}}|k) p_{t-1}(n_l^{\textrm{to}}|k)}\ , \end{aligned}$$

*M-step*

8 $$\begin{aligned} \pi _{t} (k)& = \sum _{l=1}^{L} p^{\textrm{st}}(l)r(k|l)\ , \end{aligned}$$

9 $$\begin{aligned} p_{t}(n|k) & = \frac{\alpha }{\alpha + \pi _{t}(k)} \sum _{m=1}^{N} T_{nm}p_{t-1}(m|k)\nonumber \\ & \quad+ \frac{1}{\alpha + \pi _{t}(k)} \frac{1}{2} \sum _{l=1}^{L} p^{\textrm{st}}(l)r(k|l) \left( \delta _{n,\ n_l^{\textrm{from}}} + \delta _{n,\ n_l^{\textrm{to}}} \right) \ . \end{aligned}$$

In the E-step, *r*(*k*|*l*) is the probability that link *l* belongs to community *k*; $n_l^{\textrm{from}}$ and $n_l^{\textrm{to}}$ denote the initial- and terminal-end nodes of link *l*, respectively. In the M-step, *L* is the total number of links; $\alpha$ is the only parameter whose value should be defined externally and will turn out to be controlling the resolution of community detection. See “Methods” for definitions of other notations. Pseudo code of the algorithm of MDMC is given below, where *S* and *T* are respectively the predefined numbers of steps of Markov chain and the EM-algorithm to sufficiently approach the steady states.
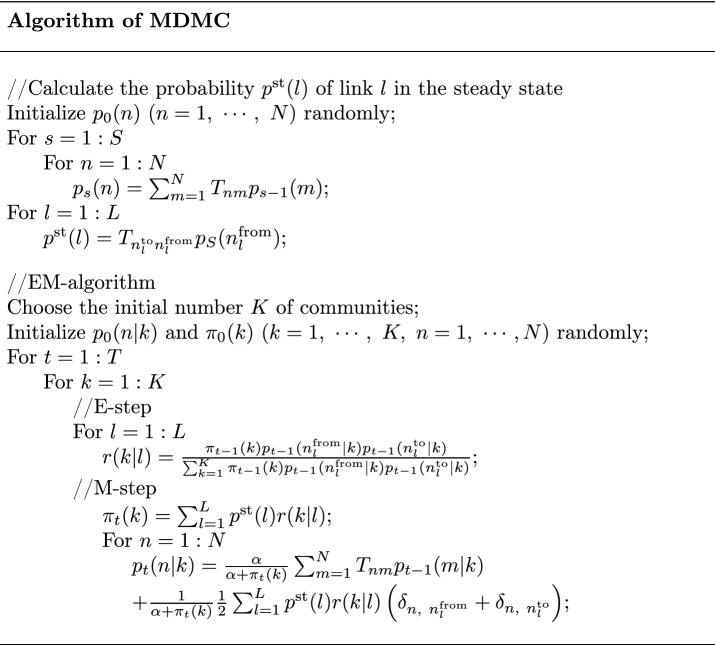


The probability that Mr. X is staying in community *k* conditioned that he is at node *n* is given by the formula

10 $$\begin{aligned} p(k|n) = \frac{p(n|k)\pi (k)}{p(n)}\ , \end{aligned}$$

which represents the relative ‘belonging’ of node *n* to community *k*. To compare communities detected by MDMC with ground-truth communities of benchmark networks, which are commonly presented as sets of non-overlapping communities called ‘partitions’^1^, we define the community to which node *n* mainly belongs by $\textrm{argmax}_k\ p(k|n)$.

### Detecting pervasive communities: fundamental properties of MDMC

**Figure 2:**
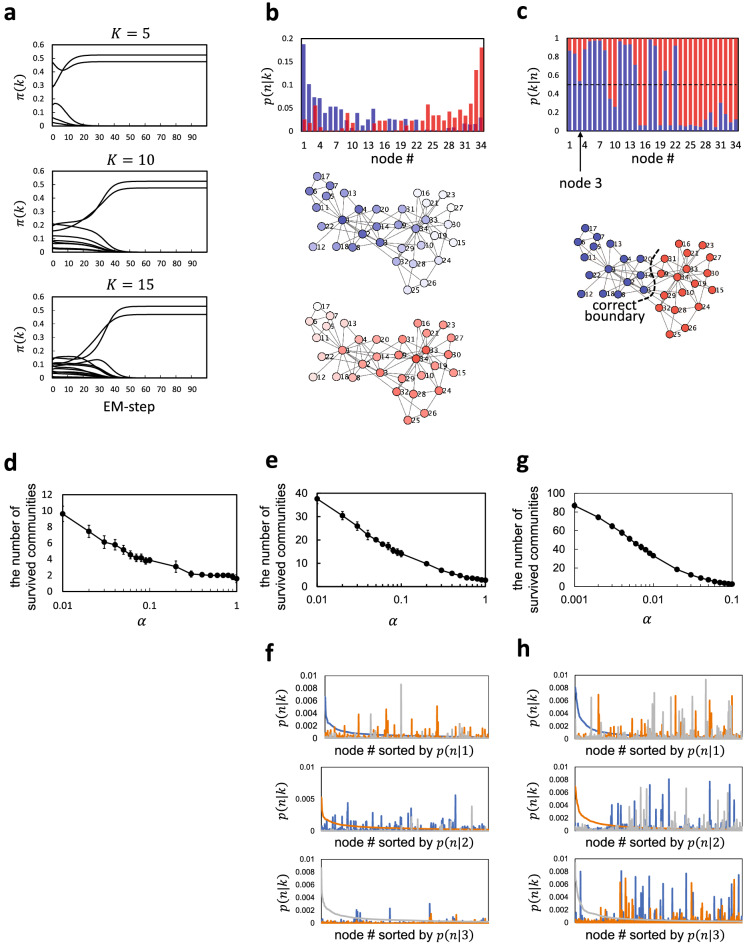
(**a**–**d**) Pervasive community detection from Zachary’s karate club network. (**a**) Evolution of community sizes $\pi (k)$ with the EM step for different settings of the putative number of communities ($K=$5, 10, and 15). (**b**) *top*, The probability distribution *p*(*n*|*k*) delineating the pervasive structure of the survived communities (indicated by blue and red); *middle* and *bottom*, Visualization of the pervasive structure of the survived communities. (**c**) *top*, The relative belonging *p*(*k*|*n*) of each node *n* to either community, with the horizontal dashed line indicating the value 0.5; *bottom*, Visualization of the main belonging of each node. (**d**) The number of survived communities, averaged over 24 trials, is plotted as a function of $\alpha$. (**e**–**h**) Pervasive community detection from larger networks: the collaboration network of condensed matter physicists (35,977 nodes and 175,692 undirected, weighted links)^47^ (**e** and **f**) and the internet peer-to-peer network (10,876 nodes and 39,994 directed links)^48^ (**g** and **h**). (**e** and **g**) The number of survived communities, averaged over 24 trials, is plotted as a function of $\alpha$. (**f**) and (**h**) The probability distributions *p*(*n*|*k*) for the three largest communities (say, communities for $k=1,\ 2$ and 3) detected from either the collaboration network for $\alpha =0.1$ (**f**) or the internet peer-to-peer network for $\alpha =0.05$ (**h**) are shown in the top, middle and bottom panels. In the top panel, the node number (#) is sorted in the abscissa according to the descending order of *p*(*n*|1) (blue). The abscissa is truncated by the top 1000 nodes just for visualization convenience. The middle and bottom panels show the same three distributions but with the node number sorted according to the descending order of *p*(*n*|2) (orange) and *p*(*n*|3) (grey), respectively. Error bars in (**d**), (**e**) and (**g**) indicate standard deviations.

First, we demonstrate fundamental properties of MDMC using Zachary’s karate club network^32,46^. Figure 2a shows how the probabilities $\pi (k)$ of communities evolve with the EM step. For any *K* chosen initially, $\pi (k)$ of only two communities survive and converge to positive values, whereas those of $K-2$ others decay to zero (for the current choice of the parameter value, here $\alpha =0.5$). Thus, MDMC automatically determines the final number of communities. Figure 2b shows the probability distributions *p*(*n*|*k*) for the survived communities, which delineate their pervasive structures. For most nodes the relative belonging *p*(*k*|*n*) to either community is near (but not exactly) unity or zero, but for node 3 the relative belongings to both communities are very close (Fig. 2c, top). Indecisive belonging of node 3 to either community accounts for why this node is often misclassified in conventional community detection^2^. Identifying the main belonging of each node by $\textrm{argmax}_k\ p(n|k)$ correctly recovers the actual separation of the karate club, which is the commonly used ground-truth of this best-known social network (Fig. 2c, bottom). We also addressed the role for parameter $\alpha$. The number of survived communities decreased as $\alpha$ increased (Fig. 2d). Thus, $\alpha$ controls the resolution of decomposition of the network into communities: For smaller $\alpha$, the network is decomposed into more communities of smaller sizes.

We also examined MDMC applied to larger networks, whose communities are supposed to have pervasive structure. Figure 2e–h shows results for the collaboration network of condensed-matter physicists (35,977 nodes and 175,692 undirected, weighted links)^47^ and the internet peer-to-peer network (10,876 nodes and 39,994 directed links)^48^. We again confirmed that the smaller the value for $\alpha$, the finer the resolution of community detection (Fig. 2e,g). The probability distributions *p*(*n*|*k*) for the three largest communities detected from either network are shown in Fig. 2f,h, which demonstrate that they are pervasively structured and formidably soft overlapping.

### Comparative study: evaluating the performance of pervasive community detection

Second, we conducted a comparative experiment to evaluate MDMC’s performance in detecting pervasive communities. The following three methods were taken for this comparison: non-negative matrix factorization (NMF)^9,10^, Bayesin non-matrix factorization (Bayesian NMF)^11^ and a stochastic block model proposed by Ball, Karrer and Newman (BKN’s SBM)^12^. Theoretically principled detection of pervasive communities is still a challenge of network science and only a few related methods have been examined so far, among which the above three are supposed to be representative. NMF tries to detect pervasive communities from a network by finding a best decomposition of its $N\times N$ adjacency matrix into the product of $N\times K$ and $K\times N$ non-negative matrices with $K (\ll N)$ being the predefined number of communities. The idea of NMF was originally inspired by Hebb’s rule^9^. Psorakis, Roberts and Ebden examined Bayesian extension of NMF (Bayesian NMF) by introducing priors^11^. Ball, Karrer and Newman^12^ proposed a stochastic block model (BKN’s SBM) that generates networks planted with pervasive communities, which can also be used conversely to detect pervasive communities from networks. Most importantly, MDMC is equivalent to BKN’s SBM for $\alpha \rightarrow 0$ (see “Methods”). In this sense, MDMC is a Bayesian extension of BKN’s SBM.

NMF, Baysian NMF, BKN’s SBM and MDMC equally assume that the network from which they try to detect communities were generated by probabilistic linking between co-appearing nodes defined by the Poisson distribution:

11 $$\begin{aligned} p\left( A_{nm} \right) = \frac{\mu ^{A_{nm}}}{A_{nm}!} e^{-\mu }\ , \end{aligned}$$

where

12 $$\begin{aligned} \mu \propto \sum _{k=1}^K \pi (k)p(n|k)p(m|k) \ . \end{aligned}$$

is the rate parameter and $A_{nm}$ is the number of links from nodes *m* to *n*. Comparing the performance of community detection between these four therefore makes sense because what they seek to find as communities are the same^2^.

**Figure 3:**
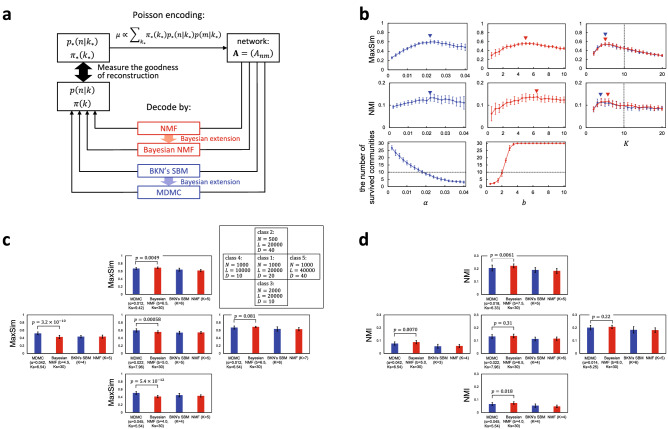
The performance of community detection from benchmark networks planted with $K_*=10$ pervasive communities was compared between MDMC, Bayesian NMF, BKN’s SBM and NMF. (**a**) Schema of our comparative experimental procedure. (**b**) Results for pervasive community detection from benchmark networks of class 1 ($N=1000$ nodes and $L=20{,}000$ undirected links, whereby the density $D=L/N=20$). MaxSim, NMI and the number of survived communities calculated by MDMC and Bayesian NMF are plotted as functions of $\alpha$ and *b* in panels of the left and centre columns, respectively. MaxSim and NMI calculated by BKN’s SBM (blue) and NMF (red) are plotted as function of the predefined number *K* in panels of the right column. Filled inverted triangles in panels of the top and middle rows indicate peak MaxSims and NMIs. Horizontal dashed lines in panels of the bottom row and vertical dashed lines in panels of the right column indicate the true number $K_*=10$ of planted pervasive communities. (**c**) Peak MaxSims are compared between MDMC, Bayesian NMF, BKN’s NMF and NMF. The centre, top, bottom, left and right panels show the results obtained for class 1 ($N=1000,\ L=20{,}000,\ D=20$), class 2 ($N=500,\ L=20{,}000,\ D=40$), class 3 ($N=2000,\ L=20{,}000,\ D=10$), class 4 ($N=1000,\ L=10{,}000,\ D=10$) and class 5 ($N=1000,\ L=40{,}000,\ D=40$) benchmark networks, respectively. (**d**) Peak NMIs are compared between the four methods. The centre, top, bottom, left and right panels show the results obtained for class 1, class 2, class 3, class 4 and class 5 benchmark networks, respectively. Error bars in (**b**–**d**) indicate standard deviations.

The schema of our comparative experiment is shown in Fig. 3a. First, probability distributions $p_*(n|k_*)$ and $\pi _*(k_*)$ $(k_*=1,\ \ldots ,\ K_*)$, which define the ‘true’ pervasive communities, are specified. Then, a benchmark network planted with these communities is generated by the Poisson distribution (11), for which BKN’s SBM is actually used. Generating a benchmark network is regarded as the process of encoding $p_*(n|k_*)$ and $\pi _*(k_*)$ into the adjacency matrix ${\varvec{A}}=\left( A_{nm} \right)$ of this network. Pervasive community detection either by NMF, Bayesian NMF, BKN’s SBM or MDMC is therefore the process of decoding the adjacency matrix into probability distributions *p*(*n*|*k*) and $\pi (k)$ $(k=1,\ \ldots ,\ K)$. The performance of pervasive community detection by each method is then evaluated by measuring how well *p*(*n*|*k*) and $\pi (k)$ reconstruct $p_*(n|k_*)$ and $\pi _*(k_*)$. Note that the number *K* of detected communities is not necessarily equal to the number $K_*$ of true communities.

Unfortunately, theoretically sophisticated measures to evaluate the goodness of pervasive community detection still have not been established. We therefore introduce a heuristic measure, the maximum similarity (MaxSim), which is given by calculating the similarity between planted $p_*(n|k_*)$ and detected *p*(*n*|*k*) by $\sum _{n=1}^N \min \left( p_*(n|k_*), p(n|k) \right)$ (see “Methods” for a detailed definition of MaxSim). Additionally, we also defined partitions (namely, clear boundaries of non-overlapping, definite communities) from planted and detected pervasive communities by calculating the main belonging of each node (by $\arg \max _{k_*}p_*(k_*|n)$ and $\arg \max _{k}p(k|n)$ for planted and detected communities, respectively); we then examined the normalized mutual information (NMI) between the ‘planted’ and ‘detected’ partitions in the expectation that NMI may serve as a measure to evaluate pervasive community detection.

MDMC has a single parameter $\alpha$ that controls the resolution of community detection. Therefore, we examined MDMC’s performance by calculating MaxSim and NMI, as well as the number of the survived communities, as functions of $\alpha$ (Fig. 3b, left column). Bayesian NMF proposed by Psorakis et al. has two parameters *a* and *b* (see “Methods”). These authors argued in the literature that: Starting with the excessive number *K* of communities, the algorithm of Bayesian NMF automatically vanishes redundant ones, as MDMC can also do so (Fig. 2a). They also argued that the number of survived communities is almost insusceptible to the values of *a* and *b*. However, we found that, at least for our benchmark networks, the number of survived communities is quite dependent especially on *b* and vanishing of redundant communities is ineffective for larger *b* (Fig. 3b, bottom of the centre column); the latter is in contrast to community detection by MDMC, where the vanishing of redundant communities is always effective though the number of survived communities varies with the resolution defined by $\alpha$ (Fig. 3b, bottom of the left column). Therefore, we examined the performance of Bayesian NMF by calculating MaxSim and NMI, as well as the number of survived communities, as functions of *b* while the value for *a* is fixed as $a=5$. The initial number of communities for MDMC and Bayesian NMF is set as $K=30$. The number of communities to which the network should be decomposed by BKN’s SBM or NMI is required to be predetermined. Therefore, we calculated MaxSim and NMI for BKN’s SBM and NMI as functions of the predefined number *K* of communities (Fig. 3b, right column).

We examined five classes of benchmark networks. Class 1 networks have the exact $N=1000$ number of nodes and the expected $L=20{,}000$ number of undirected links. We define the density by $D=L/N$. Class 1, whose density is 20, is set as a base (Fig. 3c, centre box in the inset). Class 2 and class 3 are characterized by the decreased $N=500$ and increased $N=2000$ number of nodes, respectively (Fig. 3c, top and bottom boxes in the inset). The density of class 2 and class 3 are 40 and 10, respectively. Class 4 and class 5 are characterized by the decreased $L=10{,}000$ and increased $L=40{,}000$ number of links, respectively (Fig. 3c, left and right boxes in the inset). The density of class 4 and class 5 are 10 and 40, respectively. Community detection is expected harder for the class with lower density.

For each of the five classes, 24 different benchmark networks were randomly generated to calculate MaxSim and NMI for each value of $\alpha$, *b* or *K*. Results for class 1 ($N=1000$, $K=20{,}000$ and $D=20$) are shown in Fig. 3b. MaxSim and NMI calculated by the four methods are all bell-shaped, characterized by the presence of their peak values (indicated by filled inverted triangles). Similar results obtained for the other classes of benchmark networks are shown in Supplementary Information. The number of detected communities at the peak points do not necessarily match the true number $K_*=10$ of planted communities (indicated by horizontal or vertical dashed lines in Fig. 3b).

Peak MaxSims and NMIs were compared between MDMC, Bayesian NMF, BKN’s SBM and NMF (Fig. 3c,d). Peak MaxSim by MDMC exceeds those by the other three especially for class 3 and class 4 benchmark networks, where the density is law ($D=10$). Peak MaxSims by Bayesian NMF, BKN’s SBM and NMF are almost equal. Outperforming of MDMC over Bayesian NMF is firmly statistically significant especially for class 3 and class 4 ($p=5.4 \times 10^{-12}$ and $p=3.2 \times 10^{-10}$, respectively, by Welch’s *t* test). Peak NMIs by MDMC and Bayesian NMF exceed those by BKN’s SBM and NMF for any class of benchmark networks we examined. However, we could not detect difference in peak NMI between MDMC and Bayesian NMF with firm statistical significance.

These results suggest that MDMC performs pervasive community detection better than the other three; out-performance of MDMC over the other three is remarkable especially when community detection becomes harder as the density *D* of the network becomes lower. The fact that peak NMIs by MDMC and Bayesian NMF are higher than those by BKN’s SBM and NMF suggests that Bayesian extension of BKN’s SBM and NMF may improve some aspects of community detection that can be evaluated by NMI.

We admit that our evaluation as above is unpractical, as we do not know how to optimize the parameter $\alpha$, *b* or *K* in the absence of knowledge about true communities. Indeed, to determine the optimal values for the parameters that defines the number of communities or clusters is a fundamental problem of unsupervised learning such as community detection, which is currently not fully solved. Nevertheless, the results of our evaluation suggest potential superiority of MDMC in pervasive community detection.

### System analysis of the mouse visual cortex by MDMC

**Figure 4:**
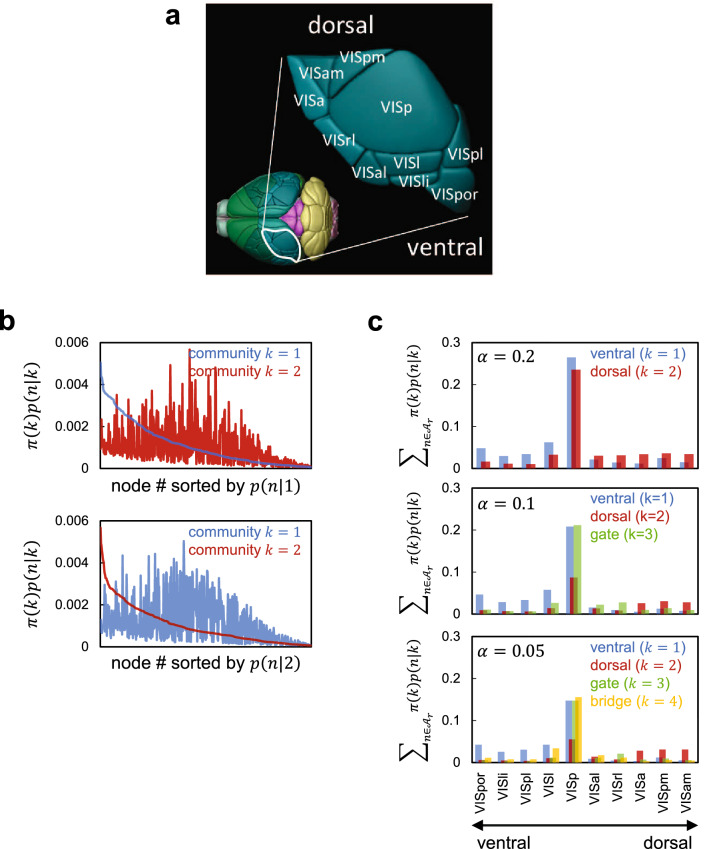
(**a**) Mouse visual cortex and its 10 areas, traced by Brain Explore (https://mouse.brain-map.org/static/brainexplorer). (**b**) The probability distributions $\pi (k)p(n|k)$ of detected two communities (say, community $k=1$ (blue) and community $k=2$ (red)) for $\alpha =0.2$. In the top and bottom panels, the node number (#) is sorted in the descending order of *p*(*n*|1) and *p*(*n*|2), respectively. (**c**) The probability distributions of communities detected from the network for $\alpha =0.2$, 0.1, and 0.05 are shown in the top, middle, and bottom panels, respectively. The ordinate value represents the probability at each area obtained by calculating $\sum _{n \in {\mathcal {A}}_{r}} \pi (k) p(n|k)$, where ${\mathcal {A}}_r$ ($r=1,\ \ldots ,\ 10$) symbolize the areas listed along the abscissa.

The pathway structure of visual cortex is known to a certain extent for humans and primates. Taking this as a putative ground truth, we examined pervasive community detection by MDMC from the structural network of mouse visual cortex (Fig. 4a), which was constructed from high-resolution connectome data^49^. The network has *N* = 468 nodes connected by *L* = 219,024 links that are directed and weighted. Each node represents a voxel and belongs to one of the 10 areas (Fig. 4a). The weight of each link represents the connection strength between the voxels connected by this link. MDMC for $\alpha$ = 0.2 detected two pervasive communities (Fig. 4b,c, top). Probability distributions of one (blue) and the other (red) communities are ventrally and dorsally biased, respectively. Both are also highly concentrated around the primary visual area (VISp). This makes sense because VISp is the gate of the visual cortex for visual sensory input, from which signals are distributed to other brain areas. The visual cortex of humans and primates is known to be functionally segregated into ventral and dorsal pathways, which are engaged in object recognition and contextual processing, respectively. Results of recent studies suggest that the visual cortex of rodents such as mice also has similar functional segregation^50–53^. Our finding that the structural network of mouse visual cortex is decomposed into pervasive communities either ventrally or dorsally biased with heavy overlapping around VISp supports this view. For $\alpha$ = 0.1, the network is decomposed into three pervasive communities (Fig. 4c, middle). The first two (blue and red) inherit the ventrally and dorsally biased communities obtained for $\alpha$ = 0.2. In the third one (green), which is seemingly separated from the dorsally biased community, most probability mass is concentrated around VISp. The third community is likely to gate the visual cortex. For $\alpha$ = 0.05, the fourth one (yellow), seemingly separated from the ventrally biased community, emerges (Fig. 4c, bottom). Besides high concentration around VISp, probability mass of the fourth community also substantially resides around VISl and VISal. We suppose that the fourth community serves as a bridge between the ventral and dorsal pathways. Interestingly, an experimental study^50^ demonstrated that a dorsal part of VISp projects to the VISl/VISal border while a ventral part projects to two separated patches (namely, VISl and VISal centres) flanking the VISi/VISal border. The former and latter projections are in agreement with the structure of the third and fourth communities, respectively.

### Hierarchical organization of pervasive communities

The observation that the resolution of decomposition into pervasive communities changes with $\alpha$ (Figs. 2d,e,g, 3b) raises a fundamental question: What is the hierarchical organization of pervasive communities? In fact, to answer this question is nontrivial. To reveal hierarchical organization of modular structure generally requires not only to find blocks (such as clusters or communities) at each layer but also to identify relationship between blocks in a given layer (say, a ‘child’ layer) and those in the next layer (its ‘parent’ layer). For hierarchical organization of definite communities, parent–child relationship can easily be identified just by tracing each node from a community with which it is affiliated in a child layer to that with which it is affiliated in the parent layer. The relationship between a community in a child layer and that in the parent layer can then be given, for instance, as the bundle of traces from the former to the latter. However, this tracing strategy is no longer effective for hierarchical organization of pervasive communities because the pervasive structure by its nature no longer defines a single or a finite number of communities to which a given node belongs.

**Figure 5:**
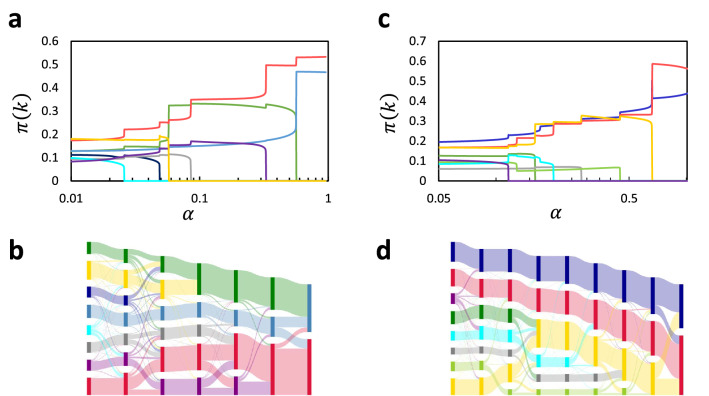
Hierarchical organization of pervasive communities of the karate club network (**a**,**b**) and the mouse whole brain network (**c**,**d**). (**a**,**c**), Each layer of hierarchy emerges through a series of discrete phase transitions induced by quasi-static increase in $\alpha$. (**b**,**d**) Sankey diagrams illustrating parent–child relationships in hierarchical organization of pervasive communities. Vertically aligned rectangles represent communities in each layer, and the width of a band connecting a child community (one rectangle in one layer) and one of its parent communities (one rectangle in the next layer) expresses the amount of flow of the probability from the former to the latter.

To address the problem, here we propose the following procedure: Fix $\alpha$ to a very small value and run the EM step to decompose the network into a large number of small communities; then, increase $\alpha$ quasi-statically (that is, very slowly) while continuing the EM step. We applied this procedure to the karate club and the mouse whole brain network^54^. For both networks we observed discrete phase transition that intermittently occurred as $\alpha$ increased (Fig. 5a,c). At each point of phase transition, the probabilities $\pi (k)$ of some communities sharply increased whereas those of some others sharply decreased (normally to zero), indicating that smaller communities merged to form larger communities. Within each interval bounded by one and the next phase transition, the probabilities $\pi (k)$ stayed almost constant, indicating a stable state corresponding to a specific layer of hierarchy.

The relationship between pervasive communities in a child layer and those in the parent layer will then be defined by flows of probabilities from the former to the latter (see “Methods” for mathematical details). The parent–child relationship thus defined can be well visualized by the Sankey diagram^55^ (Fig. 5b,d). For both networks, notably, the hierarchical organization of pervasive communities is non-tree structured; that is, a community in a child layer is generally related to more than one community in the parent layer. Especially for brain networks, the non-tree structured hierarchy indicates that distinct functional modules share or recruit the same functional sub-modules, implying an effective and flexible architecture of information processing in the brain.

It should be emphasized that serially unveiling the hierarchical organization, from layers of finer resolution to those of coarser one, by quasi-static increase in the resolution-controlling parameter $\alpha$ is essential to reveal the parent–child relationship for pervasive communities. This process enables us to trace flows of probabilities from pervasive communities in a child layer to those in the parent layer, by which we have defined the parent–child relationship. Just finding pervasive communities for different values of $\alpha$ separately is insufficient to reveal such flows. It should be noted that a similar problem is associated with extraction of hierarchical organization of definite communities, which has been sought to solve using the method of multi-layer networks^20,44^.

## Discussions

Pervasive communities, which are structurally distributed and hence characterized by the absence of clear boundaries separating their insides from their outsides, are thought to be a hallmark of biological and social networks whose cohesive structure emerges in a self-organized manner such as connecting contingently co-appearing nodes. Here we have put forward a mathematical formulation to find communities as pervasively structured objects from networks. Our formulation, which we have called modular decomposition of Markov chain (MDMC), is based on the idea that a ‘global’ random walk spreading over the entire network is expressed as a mixture of ‘local’ random walks that contribute a proxy for pervasive communities. We have applied MDMC to brain and social networks, as well as synthesized networks planted with pervasive communities, and have demonstrated that MDMC can properly detect pervasive communities from these networks.

Modularity maximization^39–44^ and map equation^33–36^ are the prevailing methods for community detection that exploit random walk, but they cannot help discover the pervasive structure of communities^1^. Moreover, they rely on greedy search such as the Louvain method^56^. By contrast, MDMC detects pervasive communities using a theoretically principled, probabilistic machine learning-based approach^14^. The computational cost of MDMC for a given resolution scales as $\sim O(KL)$, which means that MDMC belongs to the fastest class of algorithms to detect pervasive communities.

MDMC has a single parameter $\alpha$, which comes from the precision of the Dirichlet distribution describing a stochastic generalization of the Markov chain (see Eq. (3)). We have found that this parameter controls the resolution of community detection; for smaller values of $\alpha$, the network is decomposed into more communities of smaller sizes. This role of $\alpha$ is comparable with that of the time-scale parameter *t*, which appears in the modularity maximization and the map equation (see Supplementary Materials for review). To reveal hierarchical organization of pervasive communities, we propose to exploit the resolution controlling property of $\alpha$ as follows: Fix $\alpha$ to a very small value to decompose the network into many communities of small sizes; then quasi-statically increase $\alpha$ to examine the structure of pervasive communities at coarser resolution. Applying MDMC with this procedure to social and brain networks, we have observed that the hierarchy is serially unveiled from lower (finer) to upper (coarser) layers through discrete phase transitions that intermittently occurs as $\alpha$ increases. Continuously changing $\alpha$ while running MDMC is essential to reveal parent–child relationship between pervasive communities in adjacent layers. To our knowledge, this is the first elucidation of how pervasively structured modules are hierarchically organized. We believe that the proposed method is particularly beneficial to reveal the architecture of structural and functional brain networks, where cooperation of pervasiveness and hierarchy plays an essential role in flexible and adaptive information processing of the brain. Previous studies^20,57–60^, in contrast, have sought to extract hierarchical organization of definite communities from brain networks.

Graph embedding^61^ is a method to project graph objects such as nodes or links onto continuous vectors in the geometric space, which is widely used for tasks such as node classification or link prediction. Techniques of graph embedding have been developed in a research trend somewhat different from traditional network science. Interestingly, however, the-state-of-the-art methods of graph embedding also exploit random walk on the network^62,63^. Graph embedding is supposed to project nodes in the same community closely in the geometric space. With the aid of conventional clustering methods (e.g., *k*-means clustering), therefore, graph embedding could achieve community detection. Recent results suggest that graph embedding does not yield an improvement over traditional community detection algorithms as to discovery of definite, non-overlapping communities^64^. Nevertheless, as conventional machine-learning research has developed numerous methods of soft clustering, graph embedding in combination with such methods might open an avenue to pervasive community detection, which is still a challenge in traditional network science. Comparing the performance of pervasive community detection by graph embedding to that by MDMC will be conducted in future studies.

It should also be noted that MDMC itself serves as graph embedding. The conditional probabilities, $\left\{ p(k|n) \right\} _{k=1}^{K}$, can be viewed as a projection of node *n* onto a point on the simplex, $\sum _{k=1}^{K} p(k|n) =1$, embedded in the *K*-dimensional space. Alternatively, one can use a set of joint probabilities, $\left\{ p(n,\ k) \right\} _{k=1}^{K}$, as a *K*-dimensional vector representation of node *n*.

Community detection with resolution parameters^35,36,42–44^ tends to merge or split communities that are too small or too large compared to characteristic scales defined by these parameters, respectively^65–68^. We confess that MDMC with parameter $\alpha$ is also unescapable from this problem. The origin of the scale defining the size of detected communities in MDMC is the concentration of the Dirichlet prior for putative modules of random walk (see Eq. (3)). The present formulation assumes the same concentration (namely, the same $\alpha$) for each module. Our preliminary results^69^ suggest that removing this restriction remedies the resolution-limit problem. At the same time, however, we have also observed that a naive extension of the formulation confronts stiff behaviour of the EM algorithm, which results in falling into unwanted local minimums. A more mathematically-elegant formulation of MDMC to solve the resolution-limit problem will be discussed in forthcoming studies.

To establish methods for finding the true number of communities or clusters is one of the ultimate goals of unsupervised learning, which is not yet fully achieved. The present study has shown that MDMC can automatically determine the number of communities for a given value of the resolution-controlling parameter $\alpha$ (Fig. 2a), marking a step towards this goal. However, the problem of optimizing $\alpha$ still remains. In this regard, we alternatively pose a notion that: Hierarchical organization is the nature of community structure of real-world networks and the number of communities, as well as their sizes, varies depending on the resolution at which the user wants to see. For instance, decomposition of the army corps into divisions or brigades would be appropriate to strategic or tactical operations, respectively. In this view, to ask uniquely the correct number of communities may be nonsense; it would be more essential to reveal layers of hierarchy that really exist. We have demonstrated that MDMC can extract existent layers as stable states through discrete phase transitions. In physics, to be existent is to be stable. Thus, MDMC is likely to be closely approaching the solution to the problem of the number of communities. To verify the above notation, however, more extended study will be required. In particular, to solve the problem of resolution limit or detecting fals communities, which also bothers MDMC as well as standard community detection methods such as the map equation or modularity maximization, is prerequisite.

In this short article, we have focused on the theoretical foundation of MDMC and demonstrated its capability in detecting pervasive communities using a limited spectrum of networks. More extensive evaluation using a wider spectrum of networks and comparison with other community detection methods (partially conducted in Supplementary Information) will be discussed in forthcoming studies.

## Methods

### EM algorithm

In this section, we show a detailed derivation of the EM algorithm, a solver to maximization of the joint probability (6). Let ${\textbf{A}}=\left( A_{nm}\right)$ be the adjacency matrix of the network from which we wish to detect communities; $A_{nm}$ is the weight of the link from node *m* to node *n*, which can take any non-negative value. If nodes *n* and *m* are connected by an undirected link, we set $A_{nm}=A_{mn}$. The rate for transition from node *m* to node *n* is then defined by $T_{nm}=A_{nm}/\sum _{n'=1}^{N}A_{n'm}$.

According to the standard procedure to derive the EM algorithm, we first marginalize the joint probability (6) with respect to ${\textbf{Z}}$:

13 $$\begin{aligned} p\left( {\mathcal {D}},\ {\textbf{P}}_t|\left\{ \pi _t(k) \right\} _{k=1}^{K} \right) & = \sum _{{\textbf{Z}}} p\left( {\mathcal {D}},\ {\textbf{P}}_t,\ {\textbf{Z}}|\left\{ \pi _t(k) \right\} _{k=1}^{K} \right) \nonumber \\ & = \prod _{d=1}^{D} \left( \sum _{k=1}^{K} \pi _t(k) \prod _{n=1}^{N} \left[ p_t(n|k)\right] ^{\delta _{n,\ n_d^{\textrm{from}}}+\delta _{n,\ n_d^{\textrm{to}}}} \right) \nonumber \\ & \quad \times \prod _{k=1}^{K}\prod _{n=1}^{N} \left[ p_t(n|k)\right] ^{\alpha \sum _{m=1}^{N} T_{nm}p_{t-1}(m|k)}\nonumber \\ & \quad \times \prod _{k=1}^{K} \frac{\Gamma \left( \alpha + N \right) }{\prod _{n=1}^{N} \Gamma \left( \alpha \sum _{m=1}^{N} T_{nm} p_{t-1}(m|k) + 1 \right) }\ . \end{aligned}$$

Taking the log of this, we have

$$\begin{aligned} \log p\left( {\mathcal {D}},\ {\textbf{P}}_t|\left\{ \pi _t(k) \right\} _{k=1}^{K} \right) & = \sum _{d=1}^{D} \log \left( \sum _{k=1}^{K} \pi _t(k)\prod _{n=1}^{N} \left[ p_t(n|k)\right] ^{\delta _{n,\ n_d^{\textrm{from}}}+\delta _{n,\ n_d^{\textrm{to}}}} \right) \\ & \quad + \sum _{k=1}^{K}\sum _{n=1}^{N} \left( \alpha \sum _{m=1}^{N} T_{nm} p_{t-1}(m|k)\right) \log p_t(n|k)\\ & \quad + K \log \Gamma \left( \alpha + N \right) \\ & \quad - \sum _{k=1}^K \sum _{n=1}^N \log \Gamma \left( \alpha \sum _{m=1}^{N} T_{nm} p_{t-1}(m|k) + 1 \right) \ . \end{aligned}$$

Introducing $r(k|d)\ (\ge 0)$ satisfying $\sum _{k=1}^{K} r(k|d) =1$ and then using Jensen’s inequality, we derive

$$\begin{aligned}{} & {} \log \left( \sum _{k=1}^{K} \pi _t(k)\prod _{n=1}^{N} \left[ p_t(n|k)\right] ^{\delta _{n,\ n_d^{\textrm{from}}+\delta _{n,\ n_d^{\textrm{to}}}}} \right) \\{} & {} = \log \left( \sum _{k=1}^{K} r(k|d) \frac{ \pi _t(k)\prod _{n=1}^{N} \left[ p_t(n|k)\right] ^{\delta _{n,\ n_d^{\textrm{from}}}+\delta _{n,\ n_d^{\textrm{to}}}}}{r(k|d)} \right) \\{} & {} \ge \sum _{k=1}^{K} r(k|d) \log \left( \frac{ \pi _t(k)\prod _{n=1}^{N} \left[ p_t(n|k)\right] ^{\delta _{n,\ n_d^{\textrm{from}}}+\delta _{n,\ n_d^{\textrm{to}}}}}{r(k|d)} \right) \ . \end{aligned}$$

We finally obtain the lower bound *Q* of $\log p\left( {\mathcal {D}},\ {\textbf{P}}_t|\left\{ \pi _t(k) \right\} _{k=1}^{K} \right)$:

14 $$\begin{aligned} \log p\left( {\mathcal {D}},\ {\textbf{P}}_t|\left\{ \pi _t(k) \right\} _{k=1}^{K} \right) \ge Q\ , \end{aligned}$$

where

15 $$\begin{aligned} Q & = \sum _{d=1}^{D} \sum _{k=1}^{K} r(k|d) \left[ \log \pi _t(k) + \sum _{n=1}^{N} \left( \delta _{n,\ n_d^{\textrm{from}}}+\delta _{n,\ n_d^{\textrm{to}}}\right) \log p_t(n|k) - \log r(k|d) \right] \nonumber \\ & \quad + \sum _{k=1}^{K} \sum _{n=1}^{N} \left( \alpha \sum _{m=1}^{N} T_{nm} p_{t-1}(m|k)\right) \log p_t(n|k)\nonumber \\ & \quad + K \log \Gamma \left( \alpha + N \right) - \sum _{k=1}^K \sum _{n=1}^N \log \Gamma \left( \alpha \sum _{m=1}^{N} T_{nm} p_{t-1}(m|k) + 1 \right) \ . \end{aligned}$$

Maximization of the joint probability (6) can therefore be substituted with maximization of this lower bound.

Maximizing *Q* with respect to *r*(*k*|*d*) under the constrain $\sum _{k=1}^{k} r(k|d) =1$ gives the E-step:

16 $$\begin{aligned} r(k|d) & = \frac{\pi _t(k) \prod _{n=1}^{N} \left[ p_t(n|k) \right] ^{\delta _{n,\ n_d^{\textrm{from}}}+\delta _{n,\ n_d^{\textrm{to}}}}}{\sum _{k=1}^{K} \pi _t(k) \prod _{n=1}^{N} \left[ p_t(n|k) \right] ^{\delta _{n,\ n_d^{\textrm{from}}}+\delta _{n,\ n_d^{\textrm{to}}}}}\nonumber \\ & = \frac{\pi _t(k) p_t(n_d^{\textrm{from}}|k)p_t(n_d^{\textrm{to}}|k)}{\sum _{k=1}^{K} \pi _t(k) p_t(n_d^{\textrm{from}}|k)p_t(n_d^{\textrm{to}}|k)}\ . \end{aligned}$$

Maximizing *Q* with respect to $\pi (k)$ and $p_t(n|k)$ under the constraints $\sum _{k=1}^{K} \pi _t(k) =1$ and $\sum _{n=1}^{N} p_t(n|k) =1$ gives the M-step:

17 $$\begin{aligned} \pi _t(k) = \frac{D_k}{D}\ , \end{aligned}$$

18 $$\begin{aligned} p_t(n|k) & = \frac{\alpha }{\alpha +2D_k}\sum _{m=1}^{N}T_{nm}p_{t-1}(m|k)\nonumber \\ & \quad + \frac{1}{\alpha +2D_k}\sum _{d=1}^{D}r(k|d)\left( \delta _{n,\ n_d^{\textrm{from}}}+\delta _{n,\ n_d^{\textrm{to}}}\right) \ , \end{aligned}$$

where $D_k=\sum _{d=1}^{D} r(k|d)$. Note here that $p_t(n|k)$, which has been upgraded to stochastic variables by Eq. (3), is now solved by maximum a posteriori estimate.

The first and second terms on the right-hand side of Eq. (18) describe a ‘global’ random walk along common links and a bias to community *k*, respectively. The linear combination of these two as Eq. (18) therefore gives a random walk ‘localized’ to community *k*, which conforms to the idea of personalized PageRank algorithm^70,71^.

Suppose that *D* is sufficiently large. Even so, the number of observation patterns is *L*, the total number of links. Let these observation patterns be represented by $\left\{ {\tilde{\tau }}_l \right\} _{l=1}^{L}$. Here we assume that the frequency of observing pattern ${\tilde{\tau }}_l$ is approximated by $D {\tilde{p}}^{\textrm{st}}(l) = D T_{{\tilde{n}}_l^{\textrm{to}} {\tilde{n}}_l^{\textrm{from}}}p^{\textrm{st}}({\tilde{n}}_l^{\textrm{from}})$, where ${\tilde{p}}^{\textrm{st}}(l)$ and $p^{\textrm{st}}(n)$ are the probabilities of link *l* and node *n* in a stationary state of the classical Markov chain, respectively. The latter satisfies

19 $$\begin{aligned} p^{\textrm{st}}(n) = \sum _{m=1}^{N}T_{nm}p^{\textrm{st}}(m)\ . \end{aligned}$$

If the network is ergodic (that is, connected and irreducible) iterative calculation of the classical Markov chain leads to a unique stationary distribution^70^.

Therefore, we can replace the second term on the right-hand side of Eq. (18) as

20 $$\begin{aligned} \sum _{d=1}^D r(k|d) \left( \delta _{n,\ n_d^{\textrm{from}}}+\delta _{n,\ n_d^{\textrm{to}}}\right) \rightarrow D\sum _{l=1}^{L}{\tilde{p}}^{\textrm{st}}(l){\tilde{r}}(k|l)\left( \delta _{n,\ {\tilde{n}}_l^{\textrm{from}}}+\delta _{n,\ {\tilde{n}}_l^{\textrm{to}}}\right) \ , \end{aligned}$$

where ${\tilde{r}}(k|l)$ is the probability that link *l* belongs to community *k*; and ${\tilde{n}}_l^{\textrm{from}}$ and ${\tilde{n}}_l^{\textrm{to}}$ denote the initial- and terminal-end nodes of link *l*, respectively. Setting ${\tilde{\alpha }}=\alpha /2D$, we finally obtain the EM algorithm expressed by Eqs. (7), (8) and (9), where (and from now on) the ornament tildes are removed for brevity.

The *Q* function (15) is arranged in the limit $D\rightarrow +\infty$ to obtain

21 $$\begin{aligned} {\tilde{Q}}\equiv & {} \frac{Q}{2D}\nonumber \\ & = \frac{1}{2}\sum _{l=1}^{L} \sum _{k=1}^{K} {\tilde{p}}^{\textrm{st}}(l) {\tilde{r}}(k|l) \left[ \log \pi _t(k) + \sum _{n=1}^{N} \left( \delta _{n,\ n_l^{\textrm{from}}}+\delta _{n,\ n_l^{\textrm{to}}}\right) \log p_t(n|k) - \log {\tilde{r}}(k|l) \right] \nonumber \\ & \quad + {\tilde{\alpha }}\sum _{k=1}^{K} \sum _{n=1}^{N} \left( \sum _{m=1}^{N} T_{nm} p_{t-1}(m|k)\right) \log p_t(n|k)\nonumber \\ \\ & \quad - {\tilde{\alpha }} \sum _{k=1}^K \sum _{n=1}^N \left( \sum _{m=1}^{N} T_{nm} p_{t-1}(m|k) \right) \log \left( \sum _{m=1}^{N} T_{nm} p_{t-1}(m|k) \right) \ , \end{aligned}$$

where Stirling’s formula $\log z \sim \log \sqrt{2 \pi } - z + \left( z - 1/2 \right) \log z$ for $z\rightarrow +\infty$ is used. Eqs. (7), (8) and (9) can also be gained by variation of ${\tilde{Q}}$ with respect to $p_{t}(n|k)$, $\pi _t(k)$ and ${\tilde{r}}(k|l)$.

Initial conditions of the EM step are set as follows: $\pi _0(k)\ (\ge 0)$ and $p_0(n|k)\ (\ge 0)$ are chosen randomly so that $\sum _{k=1}^{K}\pi (k)=1$ and $\sum _{n=1}^{N}p_0(n|k)=1$; using these $\pi _0(k)$ and $p_0(n|k)$, we define *r*(*k*|*l*) by Eq. (7). With these initial conditions, the EM step by Eqs. (7), (8) and (9) is iterated for a predefined number of times. We can also define the convergence criteria to stop the EM step more elaborately, but in the present study we have just heuristically defined the number of iterations. Several hundred iterations are normally enough to gain aptly convergent results, but more iterations are sometimes necessary—especially when non-clique like communities are to be detected^35^.

### Teleportation: prescription for directed networks

Directed networks might include so-called ‘dangling’ nodes that have no links from them. The Markov chain for directed networks with dangling nodes no longer give ergodic stationary state because the probability is irreversibly absorbed to dangling nodes. To recover the ergodic property, we follow the prescription once proposed for the PageRank algorithm^70^. Suppose that Mr. X teleports from the current node to any node with probability $\rho$; especially when Mr. X reaches a dangling node, he teleports to any node with probability unity. These processes are implemented by replacing $T_{nm}$ with

22 $$\begin{aligned} (1-\rho )T_{nm}+(1-\rho )\frac{1}{N}\sum _{m=1}^N D_m+\rho \frac{1}{N}\, \end{aligned}$$

where $D_m=1$ if node *m* is a dangling node or $D_m=0$ otherwise. As Mr. X is unobservable during teleportation, $\sum _{l=1}^{L}p^{\textrm{st}}(l)$ becomes less than unity. To resume summed-up-to-unity, we redefine

23 $$\begin{aligned} p^{\textrm{st}}(l)\leftarrow \frac{p^{\textrm{st}}(l)}{\sum _{l=1}^{L} p^{\textrm{st}}(l)}\ . \end{aligned}$$

The above prescription is used for the mouse visual cortical network (Fig. 4) and the mouse whole brain network (Fig. 5). In both, we chose $\rho =0.15$ following the work in^70^.

### Extracting hierarchical organization of pervasive communities

MDMC has a single hyper-parameter $\alpha$. Having observed that $\alpha$ controls the resolution of decomposing the network into communities (Figs. 2d,e,g, 3b), we sought to derive hierarchical organization of communities by making use of this property. Specifically, we propose a procedure consisting of the following processes. In the first process, $\alpha$ is fixed to a very small value $\alpha _{\textrm{ini}}$ and the EM step is run to decompose the network into a large number of small communities. In the second process, $\alpha$ starts to increase quasi-statically (that is, very slowly) at time step $t_{\textrm{ini}}$ while the EM step is continued; $\alpha$ is thus increased until it reaches $\alpha _{\textrm{fin}}$ at time step $t_{\textrm{fin}}$. The resolution of decomposition is gradually reduced in the second process, whereby hierarchical structure emerged from the bottom through a series of discrete phase transitions (Fig. 3a,c). The increment of $\alpha$ per time step should be smaller for a finer resolution. To implement this, we adopted the flowing schedule for changing $\alpha$:

24 $$\begin{aligned} \alpha (t) = \alpha _{\textrm{ini}} \left( \frac{\alpha _{\textrm{fin}}}{\alpha _{\textrm{ini}}} \right) ^{(t-t_{\textrm{ini}})/(t_{\textrm{fin}}-t_{\textrm{ini}})}. \end{aligned}$$

**Figure 6:**
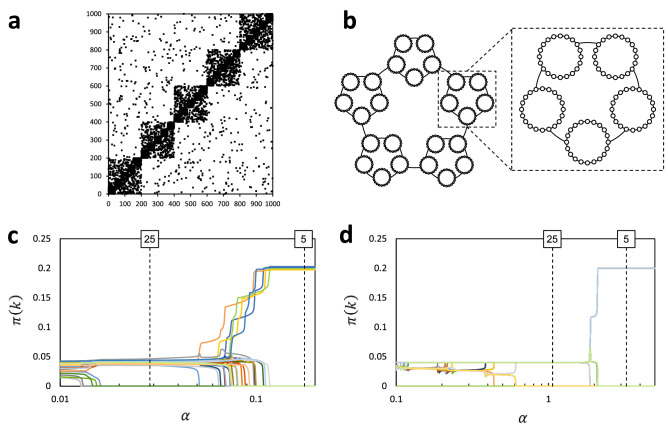
(**a**) The adjacency matrix of a block-of-blocks-of-blocks. (**b**) A network diagram of the ring-of-rings-of-rings. (**c**,**d**) The course of $\pi (k)$’s as quasi increase in $\alpha$ for the block-of-blocks-of-blocks (**c**) and the ring-of-rings-of-rings (**d**). Relatively stable phases, for which centre values for $\alpha$ are indicated by vertical dotted lines, characterize individual layers of hierarchy. A numeral attached to each dotted line indicates the number of non-vanishing $\pi (k)$’s, namely, the number of survived communities in the corresponding layer.

Before applying the above procedure to real-world networks to reveal their hierarchical organization of pervasive communities, we have examined a couple of artificial networks planted with hierarchical organization of definite communities. The first network, which we will call a ‘block-of-blocks-of-blocks’, is given by a type of Erdos-Renyi model generalized to have hierarchical organization as follows. The whole network is constructed of five sub-networks that are mutually and randomly connected; each sub-network is further constructed of five sub-sub-networks that are also mutually and randomly connected; each sub-sub-network, consisting of 40 nodes, constitutes an original Erdos-Renyi model. Its adjacency matrix is shown in Fig. 6a. The block-of-blocks-of-blocks has two layers of hierarchy. Note that each module in each layer has a cohesive structure, with its internal connections being uniformly random. The second network, which we will call a ‘ring-of-rings-of-rings’, constitutes a ring of five sub-networks; each sub-network further constitutes a ring of five sub-sub-networks; each sub-sub-network constitutes a ring of 40 nodes. The ring-of-rings-of-rings thus has two layers of hierarchy, as also seen from Fig. 6b. Each module in each layer has a non-cohesive structure. Previous studies have demonstrated that the resolution-controlling parameter plays an essential role in extracting such a non-cohesively structured communities^35^.

The above procedure was applied to the block-of-blocks-of-blocks. The obtained courses of $\pi (k)$’s with quasi-static increase in $\alpha$ are displayed in Fig 6c. We considered that relatively stable phases characterize detected layers of hierarchy. At values for $\alpha$ indicated by the vertical dotted lines in Fig 6c, therefore, we defined by using Eq. (10) definite communities constituting the corresponding layers. A numeral (‘25’ or ‘5’) attached to each vertical dotted line indicates the number of definite communities thus detected. Then, the normalized mutual information (NMI) was calculated between a set of 25 or 5 detected communities and that of 25 or 5 communities constituting the first or second layer of the planted hierarchy. Results are summaized in Table 1. The NMI is close to or exact unity in diagonal cells, while it takes much lower values in off-diagonal ones. We also applied the procedure to the ring-of-rings-of-rings and obtained essentially the same results (Fig. 6d and Table 2). These strongly support that the proposed procedure can properly extract hierarchical organization of communities.

**Table 1:** NMI between a set of detected communities and that of planted communities for the block-of-blocks-of-blocks.

	$P_{25}$	$P_5$
$D_{25}$	0.948318	0.6649
$D_5$	0.666667	1

In the most left column, $D_{25}$ and $D_5$ symbolize sets of 25 and 5 communities detected from the network for $\alpha =0.028712$ and 0.176854 (indicated by the vertical dotted lines with numerals ‘25’ and ‘5’ in Fig. 6c), respectively. In the top row, $P_{25}$ and $P_5$ symbilize sets of 25 and 5 communities constituting the first and second layers of the planted hierarchy, respectively.

**Table 2:** NMI between detected communities and planted communities for the ring-of-rings-of-rings.

	$P_{25}$	$P_5$
$D_{25}$	1	0.666667
$D_5$	0.666667	1

In the most left column, $D_{25}$ and $D_5$ symbolize sets of 25 and 5 communities detected from the network for $\alpha =1.0651$ and 3.2512 (indicated by the vertical dotted lines with numerals ‘25’ and ‘5’ in Fig. 6d), respectively. In the top row, $P_{25}$ and $P_5$ symbolize sets of 25 and 5 communities constituting the first and second layers of the planted hierarchy, respectively.

### Identifying parent–child relationships between pervasive communities

Parent–child relationships in the hierarchical organization of pervasive communities are determined in the following way. Let $p^{(h)}(k|n)$ denote the belonging of node *n* to community *k* at layer *h*, which is specifically defined by the value for *p*(*k*|*n*) at $\alpha = (\alpha _{h-1 \rightarrow h}+\alpha _{h \rightarrow h+1})/2$. Here, $\alpha _{h-1 \rightarrow h}$ is the value for $\alpha$ at which the discrete phase transition from layer $h-1$ to *h* occurred. Similarly, $\pi ^{(h)}(k)$ is defined to denote the probability of community *k* at layer *h*. The variation of *p*(*k*|*n*), the belonging of node *n* to community *k*, from layer *h* to layer $h+1$ is

25 $$\begin{aligned} \Delta p^{(h \rightarrow h+1)}(k|n)=p^{(h+1)}(k|n)-p^{(h)}(k|n)\ . \end{aligned}$$

The amounts of flow-in and flow-out of *p*(*k*|*n*) from layer *h* to layer $h+1$ are $\max \left( \Delta p^{(h \rightarrow h+1)}(k|n),\ 0\right)$ and $\max \left( -\Delta p^{(h \rightarrow h+1)}(k|n),\ 0\right)$, respectively. Therefore, the flow from $p^{(h)}(k'|n)$ to $p^{(h+1)}(k|n)$ is given by

26 $$\begin{aligned} f\left( p^{(h)}(k'|n) \rightarrow p^{(h+1)}(k|n)\right) & = \frac{\max \left( -\Delta p^{(h\rightarrow h+1)}(k'|n),\ 0 \right) }{\sum _{k'} \max \left( -\Delta p^{(h\rightarrow h+1)}(k'|n),\ 0 \right) }\nonumber \\ & \quad \times \max \left( \Delta p^{(h\rightarrow h+1)}(k|n),\ 0 \right) \ . \end{aligned}$$

Finally, the net flow from $\pi ^{(h)}(k')$ to $\pi ^{(h+1)}(k)$ is obtained by marginalizing Eq. (26) with respect to *n*:

27 $$\begin{aligned} f\left( \pi ^{(h)}(k') \rightarrow \pi ^{(h+1)}(k)\right) = \sum _{n=1}^{N} p(n) f\left( p^{(h)}(k'|n) \rightarrow p^{(h+1)}(k|n)\right) \ . \end{aligned}$$

Mergers or splits in these flows across layers can be expressed using Sankey diagrams^55^, which represent parent–child relationships in hierarchical organization of pervasive communities very well (Fig. 5b,d).

### To gain consistent structure of hierarchy

Most of community detection algorithms, including MDMC, are associated with any non-deterministic nature and their results depend on the random seeds, initial conditions or other stochastic elements. Methods to gain consistent and accurate results under such non-deterministic conditions have been devised, one of the best practices of which is the consensus clustering^19,72^.

To gain consistent structure of hierarchical organization of pervasive communities by MDMC, here we propose to refine the first process of the procedure, as follows: For a fixed value of $\alpha$ that is sufficiently small, MDMC is run many trials, with each for different random seed rendering different initial setting of the EM algorithm; the convergence value for ${\tilde{Q}}$ of Eq. (21) is calculated each trial; the random seed that gives the largest value for ${\tilde{Q}}$ is then chosen and the EM algorithm with the same fixed value for $\alpha$ is run again. The results of the first process thus optimized serve as the initial setting of the EM algorithm run in the second process with increasing $\alpha$. For a fixed number of steps between $t_{\textrm{ini}}$ and $t_{\textrm{fin}}$, the second process, which no longer involves stochastic elements, yields the consistent structure of hierarchy.

Behind the above device is our belief that the larger the convergence value for ${\tilde{Q}}$, the more accurate pervasive community detection. Additionally, the larger the number of steps between $t_{\textrm{ini}}$ and $t_{\textrm{fin}}$, which means slower increment of $\alpha$, the more accurate the structure of hierarchy at the expense of longer calculation time.

### Benchmark networks planted with pervasive communities

MDMC’s ability to detect pervasive communities was evaluated using benchmark networks planted with pervasive communities. These networks were synthesized by a specific type of stochastic block models, which has been proposed by Ball, Karrer and Newman.

#### Ball-Karrer-Newman’s stochastic block model

BKN’s SBM^12^ defines the probability of generating a network with adjacency matrix ${\textbf{A}}=\left( A_{nm}\right)$ by a Poisson distribution in the form

28 $$\begin{aligned} p\left( {\textbf{A}} \right) = \prod _{n,\ m=1}^{N} \left[ \frac{\left( \sum _{k=1}^K \theta _{nk}\theta _{mk} \right) ^{A_{nm}}}{A_{nm}!} \exp \left( -\sum _{k=1}^K \theta _{nk}\theta _{mk} \right) \right] \ . \end{aligned}$$

Here, $\theta _{nk}$ is a parameter representing the ‘propensity’ of node *n* to block *k* and taking a continuous non-negative value, whereby delineating the pervasive structure of block *k*; and $\sum _{k=1}^K \theta _{nk}\theta _{mk}$ is the rate for a Poisson event of generating a link between nodes *n* and *m*. BKN’s SBM thus generates networks planted with pervasively structured blocks (namely, pervasive communities). As $\sum _{k=1}^K \theta _{nk}\theta _{mk}$ is symmetric between *n* and *m*, BKN’s SBM is basically applicable for undirected networks. Therefore, in the rest of this section, we assume that the adjacency matrix is symmetric ($A_{nm}=A_{mn}$).

BKN’s SBM can also be used for detection of pervasive communities. This is achieved by inferring $\theta _{nk}$ for adjacency matrix ${\textbf{A}}=\left( A_{nm}\right)$ of a given network. Ball-Karrel-Newman derived the EM algorithm to solve $\theta _{nk}$, as follows:

*E-step*

29 $$\begin{aligned} q_{nm}(k) = \frac{\theta _{nk}\theta _{mk}}{\sum _{k=1}^K \theta _{nk}\theta _{mk}}\ , \end{aligned}$$

*M-step*

30 $$\begin{aligned} \theta _{nk}=\frac{\sum _{m=1}^N A_{nm}q_{nm}(k)}{\sqrt{\sum _{m=1}^N A_{nm}q_{nm}(k)}}\ . \end{aligned}$$

#### MDMC for a specific case ($\alpha$ = 0) is equivalent to BKN’s SBM

Here we demonstrate that BKN’s SBM is a specific instance of MDMC. Setting $\alpha =0$ in Eq. (9), we have

31 $$\begin{aligned} p_t(n|k) = \frac{1}{2\pi _t(k)} \sum _{l=1}^L p^{\textrm{st}}(l) r(k|l) \left( \delta _{n,\ n_l^{\textrm{from}}}+\delta _{n,\ n_l^{\textrm{to}}}\right) \ . \end{aligned}$$

For undirected networks ($A_{nm}=A_{mn}$), the steady-state distribution $p^{\textrm{st}}(n)$ can be given in the analytical form^42–44^:

32 $$\begin{aligned} p^{\textrm{st}}(n)=\frac{\sum _{m=1}^N A_{nm}}{2L}=\frac{\sum _{m=1}^N A_{mn}}{2L}\ , \end{aligned}$$

where $2L=\sum _{n,\ m=1}^{N}A_{nm}$. This leads

33 $$\begin{aligned} p^{\textrm{st}}(l)=T_{n_l^{\textrm{to}} n_l^{\textrm{from}}}p\left( n_l^{\textrm{from}}\right) = \frac{A_{n_l^{\textrm{to}} n_l^{\textrm{from}}}}{\sum _{m=1}^{N} A_{m n_l^{\textrm{from}}}} \frac{\sum _{m=1}^{N} A_{m n_l^{\textrm{from}}}}{2L}=\frac{A_{n_l^{\textrm{to}} n_l^{\textrm{from}}}}{2L}=\frac{A_{n_l^{\textrm{from}} n_l^{\textrm{to}}}}{2L}\ . \end{aligned}$$

Eq. (31) can therefore be rewritten as

34 $$\begin{aligned} p_t(n|k) = \frac{1}{2L\pi _t(k)} \sum _{m=1}^N A_{n_l^{\textrm{to}} m}r(k|l)\ . \end{aligned}$$

Now, we define $q_{n_l^{\textrm{to}} n_l^{\textrm{from}}}(k)$ and $\theta _{nk}$ in terms of *r*(*k*|*l*), $p_t(n|k)$, and $\pi _t(k)$ as

35 $$\begin{aligned}{} & {} q_{n_l^{\textrm{to}} n_l^{\textrm{from}}}(k) = r(k|l)\ , \end{aligned}$$

36 $$\begin{aligned}{} & {} \theta _{nk}=\sqrt{2L\pi _t(k)}p_t(n|k)\ . \end{aligned}$$

Rewriting Eqs. (34) and (7) in terms $q_{nm}(k)$ (of Eq. (35)) and $\theta _{nk}$ (of Eq. (36)) simply gives the EM algorithm of BKN’s SBM given by Eqs. (29) and (30).

MDMC defines the probability of observations ${\mathcal {D}}=\left\{ \tau ^{(1)},\ \ldots ,\ \tau ^{(D)}\right\}$ as

37 $$\begin{aligned} p\left( {\mathcal {D}}\right) = \prod _{d=1}^D \left[ \sum _{k=1}^K \pi (k) p(n^{(d)}|k)p(m^{(d)}|k) \right] \ . \end{aligned}$$

Suppose that the number of times at which Mr. X is observed moving along link *l* is $A_{n_l m_l}$. The result of $\sum _{n,\ m=1}^N A_{nm}=2L$ times of observation can therefore be expressed as $\left\{ A_{nm}\right\} _{n,\ m=1}^N$. Note here that $A_{nm}=0$ if nodes *n* and *m* are unconnected. From Eq. (37), we can derive the probability of $\left\{ A_{nm}\right\} _{n,\ m=1}^N$ given by a multinomial distribution in the form

38 $$\begin{aligned} p\left( {\textbf{A}} |\sum _{n,\ m=1}^N A_{nm}=2L \right) = \frac{(2L)!}{\prod _{n,\ m=1}^N A_{nm}!} \prod _{n,\ m=1}^N \left[ \sum _{k=1}^K \pi (k) p(n|k)p(m|k) \right] ^{A_{nm}}\ . \end{aligned}$$

This can be further arranged as

39 $$\begin{aligned} p\left( {\textbf{A}} |\sum _{n,\ m=1}^N A_{nm}=2L \right) & = \prod _{n,\ m=1}^N \frac{ \frac{\left[ 2L\sum _{k=1}^K \pi (k) p(n|k)p(m|k) \right] ^{A_{nm}}}{A_{nm}!} }{\frac{(2L)^{2L} \exp (-2L)}{(2L)!}}\nonumber \\ & \quad \times \exp \left( -2L\sum _{k=1}^K \pi (k) p(n|k)p(m|k) \right) \ . \end{aligned}$$

Noticing $p(\sum _{n,\ m=1}^N A_{nm}=2L)=(2L)^{2L} \exp (-2L)/(2L)!$, we finally have

40 $$\begin{aligned} p\left( {\textbf{A}}\right) & = p\left( {\textbf{A}}|\sum _{n,\ m=1}^N A_{nm}=2L\right) p(\sum _{n,\ m=1}^N A_{nm}=2L)\nonumber \\ & = \prod _{n,\ m=1}^N\frac{\left[ 2L\sum _{k=1}^K \pi (k) p(n|k)p(m|k) \right] ^{A_{nm}}}{A_{nm}!}\nonumber \\ & \quad \times {} \exp \left( -2L\sum _{k=1}^K \pi (k) p(n|k)p(m|k) \right) \ . \end{aligned}$$

Expressed in terms of $q_{nm}(k)$ and $\theta _{nk}$, this becomes Eq. (28). Thus, we conclude that MDMC for $\alpha =0$ and BKN’s SBM are equivalent. The above demonstration appears to be analogous to the proof that modularity maximization is equivalent to a specific class of stochastic block modelling^75^.

#### Benchmark networks planted with pervasive communities

Benchmark networks to evaluate pervasive community detection were synthesized using BKN’s SBM, as follows. First, $\left\{ p_*(n|k_*)\right\} _{n=1}^N$ and $\left\{ \pi _*(k_*)\right\}$ ($k_*=1,\ \ldots ,\ K_*$) were stochastically generated so that they follow power-law distributions $p\left( p_*(n|k_*)\right) \sim \left[ p_*(n|k_*)\right] ^{-\gamma }$ and $p\left( \pi _*(k_*)\right) \sim \left[ \pi _*(k_*)\right] ^{-\beta }$, respectively. Here, the subscript asterisk is used to discriminate planted ones from *p*(*n*|*k*) and $\pi (k)$ to be inferred. Powe-law distributions for $\left\{ p_*(n|k_*)\right\} _{n=1}^N$ and $\left\{ \pi _*(k_*)\right\}$ stem from the empirical fact that distributions of community sizes and degrees of nodes follow power laws in many real-world networks^73,74^. The stochastic generation of $\left\{ p_*(n|k_*)\right\} _{n=1}^N$ and $\left\{ \pi _*(k_*)\right\}$ was also devised so that the ratios $\max _n p_*(n|k_*)/\min _n p_*(n|k_*)$ and $\max _{k_*} \pi _*(k_*)/\min _{k_*} \pi _*(k_*)$ fall within moderate ranges. Benchmark networks were then generated according to the probability defined by Eq. (40). The parameter values for the synthesis were set as follows: $N=1000$, $K_*=10$, $\gamma =3$, and $\beta =2$.

#### Non-negative matrix factorization

Pervasive community detection by non-negative matrix factorizaton (NMF)^9,10^ is done by finding decomposition of the $N\times N$ adjacency matrix ${\varvec{A}}=\left( A_{nm} \right)$ into $N\times K$ and $K\times N$ non-negative matrices ${\varvec{W}}=\left( W_{nk} \right)$ and ${\varvec{H}}=\left( H_{km} \right)$ ($n,\ m=1,\ \ldots ,\ N$ and $k=1,\ \ldots ,\ K$ with $K\ll N$): ${\varvec{A}} \approx {\varvec{ WH}}$. NMF assumes network generation by the Poisson distribution $p(A_{nm})=\mu ^{A_{nm}} e^{-\mu } / A_{nm}!$ with the rate parameter $\mu \propto \sum _{k=1}^K W_{nk} H_{km}$. Therefore, ${\varvec{ W}}$ and ${\varvec{H}}$ are determined so that the generalized Kullback-Leibler divergence

41 $$\begin{aligned} D\left( {\varvec{A}} \parallel {\varvec{WH}} \right) =\sum _{n,\ m=1}^N \left( A_{nm} \log \frac{A_{nm}}{\sum _{k=1}^K W_{nk}H_{km}} -A_{nm}+\sum _{k=1}^K W_{nk}H_{km} \right) \end{aligned}$$

is maximized. Lee and Seung^10^ derived update rules to maximize this:

42 $$\begin{aligned}{} & {} W_{nk} \leftarrow W_{nk} \frac{\sum _m H_{km}A_{nm} / \sum _{k} W_{nk}H_{km}}{\sum _m H_km}, \end{aligned}$$

43 $$\begin{aligned}{} & {} H_{km} \leftarrow H_{km} \frac{\sum _m W_{nk}A_{nm} / \sum _{k} W_{nk}H_{km}}{\sum _n W_nk}. \end{aligned}$$

For undirected networks ($A_{nm}=A_{mn}$), we can set $W_{nk}=H_{km}$, whereby the relation to *p*(*n*|*k*) and $\pi (k)$ is given as

44 $$\begin{aligned}{} & {} p(n|k) = \frac{W_{nk}}{W_k} , \end{aligned}$$

45 $$\begin{aligned}{} & {} \pi (k) = \frac{W_k^2}{\sum _k W_k^2} , \end{aligned}$$

where $$W_k = \sum _n W_{nk}$$.

#### Bayesian non-negative matrix factorization

The original NMF was then extended by introducing priors^11^

46 $$\begin{aligned}{} & {} p({\varvec{ W}}|{\varvec{\beta }}) =\sum _{n} \sum _k {{\mathcal {H}}}{{\mathcal {N}}}\left( W_{nk}|0,\ \beta _k^{-1} ,\right) , \end{aligned}$$

47 $$\begin{aligned}{} & {} p(\varvec{H}|{\varvec{\beta }}) =\sum _{m} \sum _k {{\mathcal {H}}}{{\mathcal {N}}}\left( H_{km}|0,\ \beta _k^{-1} ,\right) , \end{aligned}$$

48 $$\begin{aligned}{} & {} p(\beta _k) =\frac{\beta ^{a-1}e^{-b\beta _k}b^a}{\Gamma (a)}, \end{aligned}$$

where ${{\mathcal {H}}}{{\mathcal {N}}}(x|0,\ \sigma ^2)$ denotes the half-normal distribution. Update rules for ${\varvec{W}}$, ${\varvec{H}}$ and ${\varvec{\beta }}$ are now

49 $$\begin{aligned}{} & {} W_{nk} \leftarrow \frac{W_{nk}}{\sum _m H_{km} + \beta _k W_{nk}} \frac{\sum _m H_{km}A_{nm} / \sum _{k} W_{nk}H_{km}}{\sum _m H_km}, \end{aligned}$$

50 $$\begin{aligned}{} & {} H_{km} \leftarrow \frac{H_{km}}{\sum _n W_{nk} + \beta _k H_{km}} \frac{\sum _m W_{nk}A_{nm} / \sum _{k} W_{nk}H_{km}}{\sum _n W_nk}, \end{aligned}$$

51 $$\begin{aligned}{} & {} \beta _k \leftarrow \frac{N + a -1}{\frac{1}{2}\left[ \sum _n W_{nk}^2 + \sum _m H_{km}^2 \right] }. \end{aligned}$$

For undirected networks, we can again set $W_{nk}=H_{km}$ and have the same relations (44) and (45).

#### Measuring the performance of pervasive community detection

Whereas partitioning networks into non-overlapping communities can be evaluated using information-theoretically principled measures such as normalized mutual information, sophisticated measures to evaluate pervasive community detection still have not been established. We therefore introduce a heuristic measure, the ‘maximum similarity (MaxSim)’, defined as follows. Let $K_*$ and *K* be the number of planted and detected communities, respectively. The similarity between probability distributions $p_*(n|k_*)$ and *p*(*n*|*k*) is defined by $\textrm{Sim}(k_*,\ k) =\sum _{n=1}^N \min \left( p_*(n|k_*),\ p(n|k)\right) ,$ which is then calculated for all combinations of $k_*\ (k_*=1,\ \ldots ,\ K_*)$ and $k\ (k=1,\ \ldots ,\ K)$. MaxSim is hence given by

52 $$\begin{aligned} \textrm{MaxSim}= \sum _{k=1}^{K_*} \pi _*(k_*) \left( 1-\frac{\left| \pi (k_*)-\pi (\arg \max _k \textrm{Sim}(k_*, k))\right| }{\pi (k_*)+\pi (\arg \max _k \textrm{Sim}(k_*, k))}\right) \max _{k} \textrm{Sim}(k_*, k) . \end{aligned}$$

MaxSim ranges from zero to unity. When MaxSim = 1, recovery of planted pervasive communities is perfect. For each value of $\alpha$, MDMC is examined for 24 benchmark networks (synthesized using the same parameter values but with different random seeds) and MaxSim is averaged over these networks. Pervasive community detection by BKN’s SBM is taken as a baseline for comparison. This competition seems advantageous to BKN’s SBM because the task is to detect (that is, decode) the community structure that is generated (that is, encoded) by BKN’s SBM itself. Despite that, MDMC is shown to outperform BKN’s SBM.

#### Brain network data

We believe that brain networks are the best examples of real-world networks endowed with pervasive structure of communities. Therefore, we examined two brain networks, both constructed from connectome data downloaded from Allen Mouse Brain Connectivity Atlas^49,54^. One is a structural network of mouse visual cortex, which was compiled from the high-resolution connectome data^49^. This network includes *N*=468 nodes and *L*=219,024 links. Each node represents a voxel 300 μm on each side and belongs to any one of the following 10 areas constituting the mouse visual cortex (Fig. 4a): primary visual area (VISp); lateral visual area (VISl); laterointermediate area (VISli); posterolateral visual area (VISpl); postrhinal area (VISpor); posteromedial visual area (VISpm); Anteromedial visual area (VISam); Anterior area (VISa); rostrolateral visual area (VISrl); and Anterolateral visual area (VISal). The other one is a whole brain network compiled from connectome data of the area-level resolution^54^. This network has *N* = 213 nodes and *L* = 16,954 links. Each node represents a cortical area in the right hemisphere. Links of both networks are directed and weighted.

## Supplementary Information

Supplementary Information.

## Data Availability

Data and codes used in the present study are available at: https://github.com/HO299792458/ModularDecompositionOfMarkovChain.
