# Supplementary material for: Detecting hierarchical organization of pervasive communities by modular decomposition of Markov chain
Source: Sci Rep. 2022 Nov 23;12:20211. doi: 10.1038/s41598-022-24567-x (PMC9684584; doi:10.1038/s41598-022-24567-x)
Supplement: Supplementary file 1 — Supplementary Information. [file 41598_2022_24567_MOESM1_ESM.pdf]

Supplementary Information for: Detecting hierarchical organization of pervasive communities by modular decomposition of Markov chain

Hiroshi Okamoto
okamoto@coi.t.u-tokyo.ac.jp

Xule Qiu
renaqiu1211@gmail.com

1 What does MDMC decompose?

Here we demonstrate that $p(n)$ to be decomposed by MDMC is well approximated by $p^{\mathrm{st}}(n)$, the stationary-state distribution of the Markov chain. Given that the EM step leads to a stationary state, Eq. (9) in the main text is rewritten as

$$p(n|k) = \frac{\alpha}{\alpha + \pi(k)} \sum_{m=1}^{N} T_{nm} p(m|k) + \frac{\pi(k)}{\alpha + \pi(k)} p_0(n|k) \ , \quad (1)$$

where

$$p_0(n|k) = \frac{1}{2\pi(k)} \sum_{l=1}^{L} p^{\mathrm{st}}(l) r(k|l) \left(\delta_{n,\ n_l^{\mathrm{from}}} + \delta_{n,\ n_l^{\mathrm{to}}} \right) \ . \quad (2)$$

Using the property $\sum_{k=1}^{K} \pi(k) p_0(n|k) = p^{\mathrm{st}}(n)$, one derives

$$p(n) - p^{\mathrm{st}}(n) = \alpha \sum_{k=1}^{K} \left[\sum_{m=1}^{N} T_{nm} p(m|k) - \sum_{k=1}^{K} p(n|k) \right] \ . \quad (3)$$

If $\alpha = 0$ is small relative to $\pi(k)$'s, one straightforwardly has $p(n) \approx p^{\mathrm{st}}(n)$. On the other hand, if α is relatively large, Eq. (1) can be arranged in the first-order approximation to $\pi(k)/\alpha$ as

$$\frac{\pi(k)}{\alpha} \left[\sum_{m=1}^{N} T_{nm} p(m|k) - p_{\alpha=0}(n|k) \right] = \sum_{m=1}^{N} T_{nm} p(m|k) - p(n|k) \ . \quad (4)$$

Summing these over k's and then using the exact relation (3), one obtains $\sum_{m=1}^{N} T_{nm} p(m) = p(n)$, from which the uniqueness of a stationary-state solution of the Markov chain, therefore, gives $p(n) = p^{\mathrm{st}}(n)$. We have confirmed that for the overall range of α, $p(n) \approx p^{\mathrm{st}}(n)$ holds for a variety of networks (Fig. 4). Thus, we conclude that MDMC accurately decomposes $p^{\mathrm{st}}(n)$ into modules as a proxy for communities.

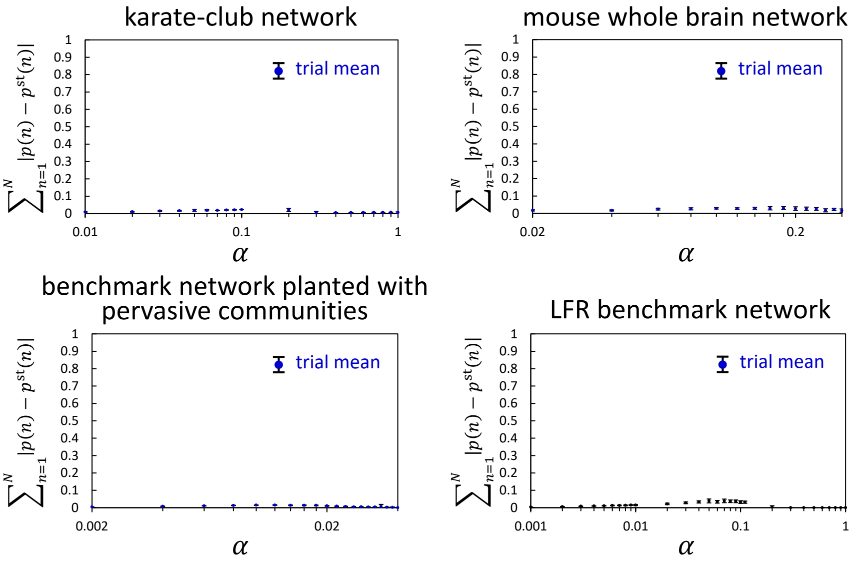

Figure 1: The difference between $p(n)$ and $p^{\text{st}}(n)$ is estimated by $\sum_{n=1}^{N} |p(n) - p^{\text{st}}(n)|$ for a variety of networks: *top left,* the karate club network; *top right,* the mouse whole brain networks (directed and weighted); *bottom left,* a benchmark network planted with pervasive communities (1000 nodes); *bottom right,* a Lancichinetti–Fortunato–Radicchi benchmark network (1000 nodes) [1]. In each panel, the difference averaged over 12 trials is plotted as a function of α. For any network at any value of α, the difference remains less than 0.05.

2 Random-walk formulations of the map equation and the modularity maximization

The map equation [2, 3] and the modularity maximization [4, 5, 6] of resolution controlling versions can be formulated in the framework of random walk [7, 8, 9, 10, 11]. which serve as baselines in the present study. For readers' convenience, we briefly review these formulations below.

2.1 Map equation with resolution control

The map equation finds communities by searching for the most parsimonious way to describe a random walk taking place on the network. The map equation of a resolution-controlling version [7, 8] can be derived from the continuous-time random-walk dynamics, which is described by the master equation

$$\frac{dp_n(t)}{dt} = -p_n(t) + \sum_{m=1}^{N} T_{nm} p_m(t) \ . \tag{5}$$

The formal solution to this is

$$p_n(t) = \sum_{m=1}^{N} T^{(\mathrm{C})}_{nm}(t) p_m(0) \ , \tag{6}$$

where

$$T^{(\mathrm{C})}_{nm}(t) = \left(e^{-t(\mathbf{I}-\mathbf{T})} \right)_{nm} \tag{7}$$

is the probability of moving from nodes m to n in an interval of the length t; $\mathbf{T} = (T_{nm})$ and $\mathbf{I} = (\delta_{nm})$ are the transition rate matrix and the identity matrix, respectively.

The map equation defines the description length for coding a random walk on the network. Suppose that a random walk is partitioned into K groups at a timescale characterized by t. According to the Shannon theorem, the map equation is given as a weighted combination of the entropies in the form

$$L_M(t) = \sum_{k=1}^{K} p^k_{\circlearrowright}(t) H\left(\mathcal{P}^k(t)\right) + q_{\curvearrowleft}(t) H\left(\mathcal{Q}(t)\right) \ , \tag{8}$$

where

$$H\left(\mathcal{P}^k(t)\right) = -\frac{q_{k\curvearrowright}(t)}{p^k_{\circlearrowright}(t)} \log_2 \left(\frac{q_{k\curvearrowright}(t)}{p^k_{\circlearrowright}(t)} \right) - \sum_{n \in C_k} \frac{p_n(t)}{p^k_{\circlearrowright}(t)} \log_2 \left(\frac{p_n(t)}{p^k_{\circlearrowright}(t)} \right) \ , \tag{9}$$

$$H\left(\mathcal{Q}(t)\right) = -\sum_{k=1}^{K} \frac{q_{k\curvearrowleft}(t)}{q_{\curvearrowleft}(t)} \log_2 \left(\frac{q_{k\curvearrowleft}(t)}{q_{\curvearrowleft}(t)} \right) \ . \tag{10}$$

Here, $q_{k\curvearrowright}(t)$ and $q_{k\curvearrowleft}(t)$ are the probabilities of leaving and arriving group k $(= 1, \ \cdots, \ K)$ within t, respectively, and are defined as

$$q_{k\curvearrowright}(t) = \sum_{n \notin C_k} \sum_{m \in C_k} T^{(\mathrm{C})}_{nm}(t) p_m(0) \ , \tag{11}$$

$$q_{k\curvearrowleft}(t) = \sum_{n \in C_k} \sum_{m \notin C_k} T^{(\mathrm{C})}_{nm}(t) p_m(0) \ . \tag{12}$$

The $q_{\curvearrowright}(t)$ and $q_{\curvearrowleft}(t)$ are the probabilities of leaving and arriving any group within t, respectively, and are defined as

$$q_{\curvearrowleft}(t) = \sum_{k} q_{k\curvearrowleft}(t) \ , \quad q_{\curvearrowright}(t) = \sum_{k} q_{k\curvearrowright}(t) \ . \tag{13}$$

Since exact calculation of Eq. (6) is infeasible especially for large networks, the following approximation is adopted [7, 8]:

$$\mathbf{T}^{(\mathrm{C})}(t) = \begin{cases} (1-t)\mathbf{I} + t\mathbf{T} & (t < 1) \ , \\ t\mathbf{T} & (t \geq 1) \ . \end{cases} \tag{14}$$

Among all partitions, the one that minimizes the map equation (8) defines the decomposition of the network into communities. The above formulation has a single parameter t, which controls the resolution of community detection: For larger values of t, the network is decomposed into a smaller number of larger communities.

2.2 Modularity maximization with resolution control

The resolution controlling version of the modularity maximization can also be derived from the continuous-time random-walk dynamics [9, 10, 11]. For a given partition with K groups, the probability that a random walker remains in the same community within an interval of length t, relative to that expected under randomization in the equilibrium, is given by

$$R(t) = \sum_{n,\ m} \left[\left(e^{-t(\mathbf{I}-\mathbf{T})} \right)_{nm} p_m - p_n p_m \right] \delta\left(g_n,\ g_m\right) \ , \tag{15}$$

where g_n represents the group to which node n belongs; $\delta(g_n,\ g_m) = 1$ if nodes n and m both belong to the same group and it is zero otherwise; p_n is the steady state solution of the master equation (5). Expanding $e^{-t(\mathbf{I}-\mathbf{T})}$ to the first-order in t yields

$$R(t) = \sum_{n,\ m} \left(tT_{nm} p_m - p_n p_m \right) \delta\left(g_n,\ g_m\right) - t \ . \tag{16}$$

We hence define

$$Q(t) = \frac{R(t) + t}{t} = \sum_{n,\ m} \left(T_{nm} p_m - \frac{1}{t} p_n p_m \right) \delta\left(g_n,\ g_m\right) \ . \tag{17}$$

In the case of undirected, binary networks where $A_{nm} = 1$ (connected) or 0 (disconnected), $T_{nm} = A_{nm}/k_m$ with k_m being the degree of node m, and the steady state solution is trivially given by $p_n = k_n/2L$. We therefore have

$$Q(t) = \frac{1}{2L} \sum_{n,\ m} \left(A_{nm} p_m - \frac{1}{t} \frac{k_n k_m}{2L} \right) \delta\left(g_n,\ g_m\right) \ . \tag{18}$$

When $t = 1$, Eq (18) gives classical Newman's modularity. Communities are sought by searching for the partition that maximizes Q, or equivalently, the relative probability (15). The only parameter t controls the resolution of community detection: For larger values of t, the network is decomposed into a smaller number of larger communities.

2.3 Louvain method

Since finding from all possible partitions the one that globally maximizes the modularity is NP hard, a heuristic approach, called the Louvain method [13],

is generally used. This executes a greedy maximization of the modularity in an aggregatory manner: At first, each node is assigned to a group that has this node as a single member; then, a randomly chosen pair of neighbouring groups are aggregated to form a larger group if this aggregation increases the modularity; updating the partition by this way is continued until aggregation of any pair of neighbouring groups no more increases the modularity. Groups of the partition thus obtained are defined as communities of the network. This greedy approach by no means ensures the global maximum, yet it has exhibited high performance of community detection in comparative studies. Minimization of the map equation is also achieved by the Louvain method in the same way except that decrease, but not increase, in the evaluation function (map equation) allows aggregation of a pair of neighbouring groups.

2.4 Supplemental data of comparative experimental results

Supplemental data of results of the comparative experiment (Fig. 3 in the main text) are shown in Fig. S2.

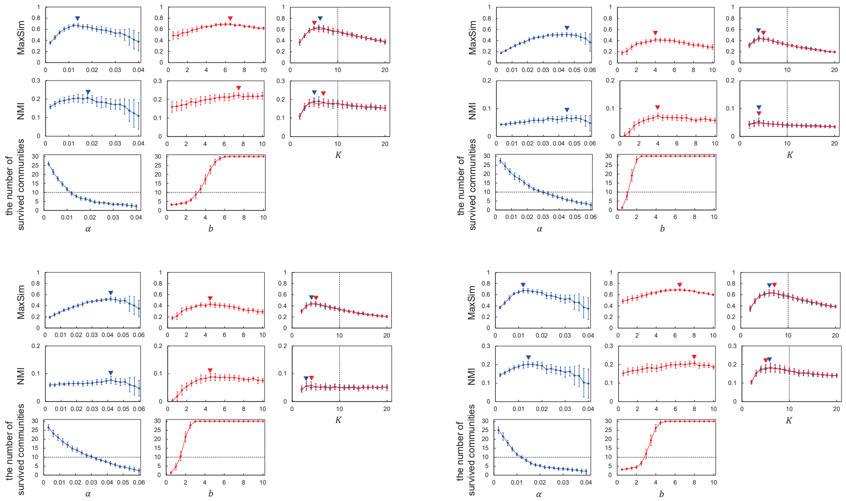

Figure 2: Results of the comparative experiment to evaluated the performance of pervasive community detection. Evaluation was conducted using five classes of benchmark networks. Results for class 1 benchmark networks are shown in Fig. 3b of the main text. Results for the other classes are shown here. **a**, Class 2 ($N = 500$, $L = 20000$, $D = 40$). **b**, Class 3 ($N = 2000$, $L = 20000$, $D = 10$). **a**, Class 4 ($N = 1000$, $L = 10000$, $D = 10$). **b**, Class 5 ($N = 1000$, $L = 40000$, $D = 40$).

2.5 MDMC's performance in partition detection

The map equation and the modularity maximization, even in their resolution-controlling versions, detect partitions, that is, boundaries of non-overlapping definite communities from networks. MDMC, originally designed to detect communities as pervasively structured objects, can also achieve definite community detection by examining $\arg\max_k p(k|n)$ as described in the text. We therefore evaluated definite community detection by MDMC taking the map equation and the modularity maximization of resolution controlling versions as baselines. We conducted this evaluation using a variety of networks with ground truths; for instance, Zachary's karate club [14], American College football [15], Books about US politics [16], Lancichinetti–Fortunato–Radicchi benchmark networks [1] of mixed parameters $\mu = 0.1$, 0.3 and 0.5. The normalized mutual information (NMI) between detected communities and the ground-truth ones was calculated as a function of the resolution parameter (α for MDMC and t for the map equation and the modularity maximization) . The results shown in Fig. 3 demonstrate that MDMC exhibits the same level of performance as the map equation and the modularity maximization, which are the best or most standard methods for definite community detection.

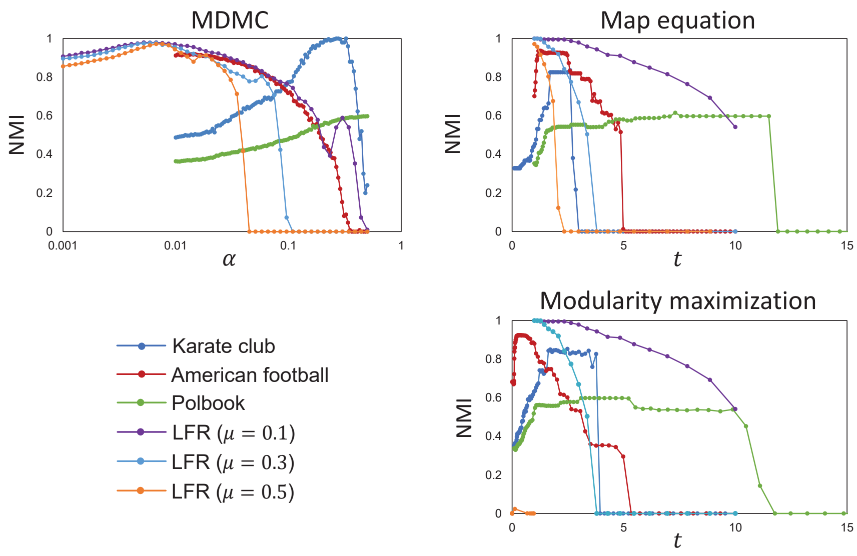

Figure 3: Definite community detection by MDMC (*top left*), the map equation (*top right*) or the modularity maximization (*bottom right*) from a variety of networks with ground truths. The normalized mutual information (NMI) between detected communities and ground-truth ones is plotted as a function of the resolution parameter (α for MDMC and t for the map equation and the modularity maximization). Karate club, Zachary's karate club; American football, American college football; Polbook, Books about US politics; LFR, Lancichinetti–Fortunato–Radicchibenchmark networks.

References

[1] Lancichinetti, A., Fortunato, S., Radicchi. F. Benchmark graphs for testing community detection algorithms. Phys Rev E 78, 046110 (2008)

[2] Rosvall, M., Bergstrom, C.T. An information-theoretic framework for resolving community structure in complex networks. Proc Natl Acad Sci USA 104: 7327–7331 (2007)

[3] Rosvall, M., Bergstrom, C.T. Maps of random walks on complex networks reveal community structure. Proc Natl Acad Sci USA 105: 1118–1123 (2008)

[4] Newman, M.E.J. Modularity and community structure in networks. Proc Natl Acad Sci USA 103, 8577–8582 (2006)

[5] Newman, M.E.J. Fast algorithm for detecting community structure in networks. Phys Rev E 70, 066133 (2006)

[6] Newman, M.E.J. Communities, modules and large-scale structure in networks. Nat Phys 8, 25–31 (2012)

[7] Schaub, M.T., Lambiotte, R., Barahona, M. Encoding dynamics for multiscale community detection: Markov time sweeping for the map equation. Phys Rev E 86, 026112 (2012)

[8] Kheirkhahzadeh, M., Lancichinetti, A., Rosvall M. Efficient community detection of network flows for varying Markov times and bipartite networks. Phys Rev E 93, 032309 (2016)

[9] Lambiotte, R., Delvenne, J.C., Barahona, M. Laplacian dynamics and multiscale modular structure in networks. arXiv:0812.1770v3 (2009)

[10] Delvenne, J.C., Yaliraki, S.N., Barahona, M. Stability of graph communities across time scales. Proc Natl Acad Sci USA 107, 12755–12760 (2010)

[11] Mucha, P.J., et al. Community structure in time-dependent, multiscale, and multiplex networks. Science 328, 876–878 (2010)

[12] Masuda, N., Poretr, M.A., Lambiotte, R. Random walks and diffusion on networks. Phys Rep 716-717, 1–58 (2017)

[13] Blondel, V.D., et al. Fast unfolding of communities in large networks. J Stat Mech, P10008 (2008)

[14] Zachary, W.W. An information flow model for conflict and fission in small groups. J Anthro Res 33, 452–473 (1977)

[15] Girvan, M., Newman, M.E.J. Community structure in social and biological networks. Proc Natl Acad Sci USA 99, 7821–7826 (2002)

[16] Adamic, L.A., Glance, N. The political blogosphere and the 2004 US Election: in Proceedings of the WWW-2005 Workshop on the Weblogging Ecosystem (2005)
